# Epigenetic changes in myelofibrosis: Distinct methylation changes in the myeloid compartments and in cases with *ASXL1* mutations

**DOI:** 10.1038/s41598-017-07057-3

**Published:** 2017-07-28

**Authors:** Helene Myrtue Nielsen, Christen Lykkegaard Andersen, Maj Westman, Lasse Sommer Kristensen, Fazila Asmar, Torben Arvid Kruse, Mads Thomassen, Thomas Stauffer Larsen, Vibe Skov, Lise Lotte Hansen, Ole Weis Bjerrum, Hans Carl Hasselbalch, Vasu Punj, Kirsten Grønbæk

**Affiliations:** 1 Department of Hematology, Rigshospitalet, Copenhagen University Hospital, Copenhagen, Denmark; 2 Department of Biomedicine, Aarhus University, Aarhus, Denmark; 3 Danish Stem Cell Centre (DanStem) Faculty of Health Sciences, University of Copenhagen, Copenhagen, Denmark; 4 Department of Hematology, Roskilde Hospital, Roskilde, Denmark; 5 Department of Clinical Genetics, Rigshospitalet, Copenhagen, Denmark; 6 Department of Clinical Genetics, Odense University Hospital, Odense, Denmark; 7 Department of Hematology, Odense University Hospital, Odense, Denmark; 8 Division of Hematology, Keck School of Medicine, University of Southern California, Los Angeles, United States

## Abstract

This is the first study to compare genome-wide DNA methylation profiles of sorted blood cells from myelofibrosis (MF) patients and healthy controls. We found that differentially methylated CpG sites located to genes involved in ‘cancer’ and ‘embryonic development’ in MF CD34+ cells, in ‘inflammatory disease’ in MF mononuclear cells, and in ‘immunological diseases’ in MF granulocytes. Only few differentially methylated CpG sites were common among the three cell populations. Mutations in the epigenetic regulators *ASXL1* (47%) and *TET2* (20%) were not associated with a specific DNA methylation pattern using an unsupervised approach. However, in a supervised analysis of *ASXL1* mutated versus wild-type cases, differentially methylated CpG sites were enriched in regions marked by histone H3K4me1, histone H3K27me3, and the bivalent histone mark H3K27me3 + H3K4me3 in human CD34+ cells. Hypermethylation of selected CpG sites was confirmed in a separate validation cohort of 30 MF patients by pyrosequencing. Altogether, we show that individual MF cell populations have distinct differentially methylated genes relative to their normal counterparts, which likely contribute to the phenotypic characteristics of MF. Furthermore, differentially methylated CpG sites in *ASXL1* mutated MF cases are found in regulatory regions that could be associated with aberrant gene expression of ASXL1 target genes.

## Introduction

The chronic myeloproliferative neoplasms (MPNs) include the classical diseases myelofibrosis (MF), polycythemia vera (PV), and essential thrombocythemia (ET), with MF patients having the highest morbidity and mortality^1^. In addition to the expansion of one or more of the myeloid lineages, MF is characterized by progressive bone marrow fibrosis leading to extramedullary hematopoiesis and hepatosplenomegaly^2^.

The most commonly observed mutation in MF is *JAK2*V617F, which is found in 60% of MF patients^3^. Eight to 11% of *JAK2*V617F negative MF patients carry *MPL* mutations^4^, and both *JAK2* and *MPL* mutations cause constitutive activation of the JAK/STAT pathway that promotes cell survival and proliferation^5^. More recently, mutations were identified in *CALR* that are mutually exclusive to *JAK2* and *MPL* mutations in the majority of patients^6, 7^. In addition to causing constitutive activation of the JAK/STAT pathway^7^, mutated CALR lose the ability to bind calcium and retrieve and retain chaperone proteins to the endoplasmic reticulum^6, 7^. Although mutations in *JAK2*, *MPL*, and *CALR* are recurrent in MPN, they alone explain neither the pathogenesis nor the clinical manifestations associated with the distinctive MPN subgroups.

Mutations in epigenetic regulators, including *ASXL1*, *TET2*, *DNMT3A*, *EED*, *EZH2*, *IDH1/2*, *JARID2*, and *SUZ12* have also been observed in MF^8, 9^, and expansion of the *ASXL1* mutated clone has been associated with leukemic transformation^10^. However, despite high frequency of mutations in some of these genes, little is known about their impact on epigenetic regulation in MF. Few studies have investigated the genome-wide methylation patters in MF^11, 12^, and none of them have compared different MF cell populations.

Both *TET2* and *ASXL1* mutations have been associated with increased DNA methylation levels when analyzing neutrophils^11^, and unsorted cells from bone marrow and peripheral blood^12^. In addition, *ASXL1* mutations were associated with a distinct DNA methylation signature^11^. Disruption of *ASXL1* is frequent in myeloid malignancies with a prevalence of 20–30%^13–15^. *In vivo* analysis shows that hematopoiesis-specific loss of *Asxl1* causes multi lineage cytopenia and dysplasia^13^ indicating its pivotal role in hematopoiesis. ASXL1 and BAP1 constitute a deubiquitination complex, where BAP1 catalyze the deubiquitination of H2AK119Ub^16, 17^. H2AK119Ub is a repressive histone mark deposited by the Polycomb Repressive Complex 1 (PRC1)^18^, both in a PRC2-dependent^19^ and independent manner^20^. Moreover, it was recently observed that H2AK119Ub could recruit components of the PRC2 complex to catalyze H3K27me3^21^.

Since MF is a disease affecting several hematopoietic cell lineages, we investigated the genome-wide DNA methylation profiles of CD34+ cells, mononuclear cells, and granulocytes from 16 MF patients and 3 healthy age-matched controls. We further aimed to investigate whether distinct DNA methylation profiles are related to genetic aberrations of any of the epigenetic modifiers *ASXL1*, *TET2*, *DNMT3A*, *IDH1*, and *IDH2*.

## Results

By comparison of individual MF cell populations to their normal counterparts isolated from healthy donors, we initially identified differentially methylated CpG sites within MF granulocytes, MF mononuclear cells and MF CD34+ cells, respectively.

### MF granulocytes are hypomethylated relative to MF CD34+ cells and MF mononuclear cells

Based on the 504 most differentially methylated CpG sites with a standard deviation (SD) > 0.3 across all samples, a hierarchical cluster analysis clearly distinguished individual samples of MF mononuclear cells and MF CD34+ cells from MF granulocytes (Figure S1). In general, granulocytes were characterized by an overall low methylation level, which correlates to previous findings^22^. A single MF granulocyte sample (F16) clustered together with the mononuclear cells and CD34+ cells due to a higher overall methylation level. Three distinct clusters were observed in the granulocyte population; however, this could not be explained by mutations in any of the genes investigated.

### Each MF cell compartment has a specific DNA methylation profile

**Figure 1:**
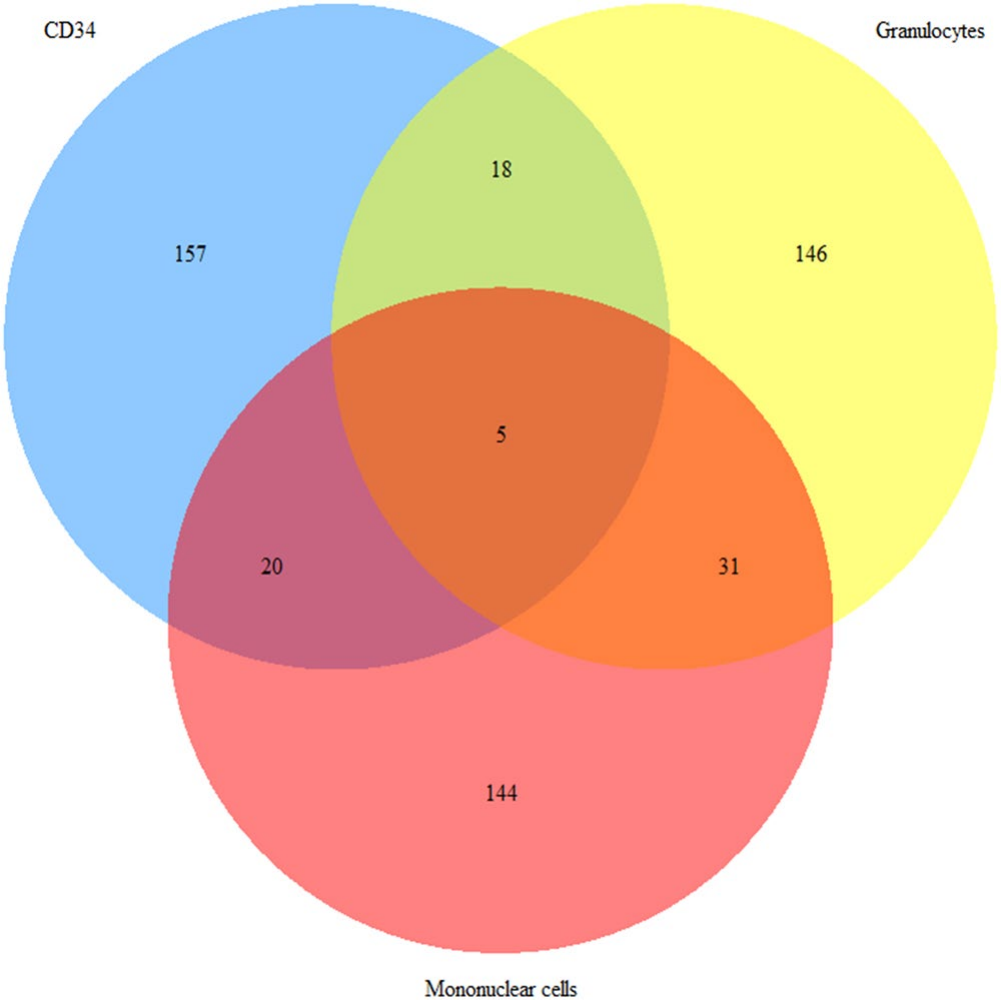
Venn diagram showing the overlap of differentially methylated CpG sites between MF cell populations. The CD34+ cell population is blue, the MF granulocyte population is yellow, and the MF mononuclear cell population is red. Five differentially methylated CpG sites overlapped between the three cell populations.

A Venn diagram was used to illustrate the overlap of differentially methylated CpG sites between the MF granulocytes, MF mononuclear cells and MF CD34+ cells. The 200 most significantly differentially methylated CpG sites were included, and only a minor overlap was observed between the three MF cell populations (Fig. 1).

### Aberrantly methylated genes in the MF CD34 + cell population

In the MF CD34+ cells, 1628 CpG sites annotated to 739 genes were differentially methylated (FDR p < 0.05; |Δβ| ± 0.2) when compared to their healthy counterparts (Table S2). Ingenuity pathway analysis revealed that differentially methylated CpG sites were annotated to genes involved in ‘cancer’ (e.g. *WT1*, *BCL2*, *BIN1*, *GATA6*, *RUNX2*, *EGFR*) and ‘embryonic development’ (e.g. *WT1*, *BMP4*, *FOXC1*, *GATA4*), ‘cell death and survival’ (e.g. *BCL2*, *EGFR*, *BMP4*) ‘hematopoiesis’ (e.g. *BMP4*), ‘cell cycle’ (e.g. *EGFR*, *BMP4*), and ‘hematological diseases’(e.g. *JAK2*) (Figure S2A).

### Aberrantly methylated genes in the MF mononuclear cells

In MF mononuclear cells, 213 CpG sites annotated to 121 genes were differentially methylated (FDR p < 0.05; |Δβ| ± 0.2) when compared to their healthy counterparts (Table S3). Ingenuity pathway analysis revealed that differentially methylated CpG sites were annotated to genes involved in ‘cell cycling’ (e.g. *NDRG1*, *NEDD1*, and *MAD1L1*), ‘inflammatory diseases’ (e.g. *PRTN3*), and ‘cancer’ (*PCDHA6*, *MUC4*, and *ATP2C2*) (Figure S2B).

### Aberrantly methylated genes in the MF granulocyte population

In the MF granulocytes, 519 CpG sites annotated to 303 genes were differentially methylated (FDR p < 0.05; |Δβ| ± 0.3) when compared to their healthy counterparts (Table S4). Ingenuity pathway analysis revealed that differentially methylated CpG sites were annotated to genes involved in ‘cancer’ (e.g. *WT1*, *BIN1*, *PCDHA6*, *RIPK4*, *SOCS3*, *KTN1*), ‘cellular growth and proliferation’ (e.g. *mir-146*, *WT1*, *FOXP1*, *CEBPE*, *IGF2BP1*, *IGF1R*, *CASP8*), ‘immunological diseases’ (e.g. *BCL2L1* and *MICA*) and in ‘cell death and survival’ (e.g. *SOCS3*, *mir-146*, *UHRF1*, and *CASP8*) (Figure S2C).

### Investigation of differentially methylated CpG sites in the validation cohort

**Figure 2:**
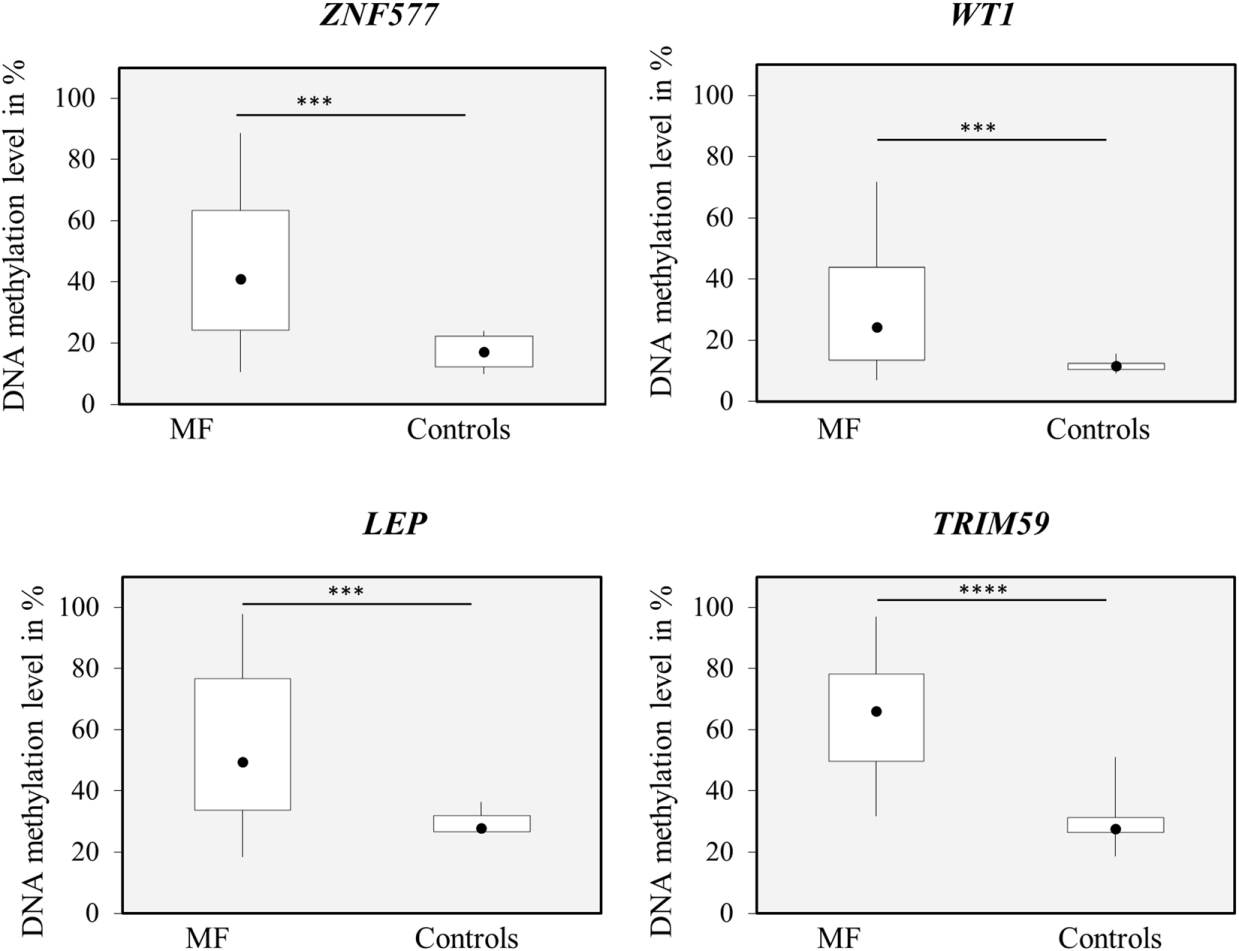
Validation of the methylated genes in a validation MF cohort. The DNA methylation level of 2–8 CpG sites annotated to four genes (*ZNF577*, *WT1*, *LEP*, and *TRIM59*) was validated in a validation MF cohort consisting of 30 MF patients where DNA had been isolated from whole blood. Hypermethylation of the *ZNF577*, *LEP*, and *TRIM59* promoter regions and the *WT1* gene body was verified using pyrosequencing (P ≤ 0.001 for all genes analyzed, Mann-Whitney test).

We selected four genes (*LEP*, *TRIM59*, *WT1*, and *ZNF577*) with at least two differentially methylated CpG sites in close proximity to the transcription start site for further validation. Differential methylation was confirmed using pyrosequencing for all genes in the MF validation cohort comprising 30 MF whole blood samples compared to 11 whole blood samples from healthy individuals (Fig. 2).

### Somatic mutations in the MF cases

The mutational status of *JAK*2 was determined for all 16 MF patients (Table 1), whereas the mutational status of *TET2*, *ASXL1*, *DNMT3A*, *IDH1*, *IDH2*, *CALR*, and *MPL* was only determined in 15 patients due to limited material (Table 2). The most frequent mutations in the epigenetic regulators were nonsense mutations predicted to cause premature termination in *ASXL1*, which was observed in six patients (no. 1, 3, 5, 10, 14, and 15). Patient 7 had a missense mutation in *ASXL1* causing the p.N986S substitution. Truncating mutations in the *TET2* gene were observed for three patients (no. 1, 5, and 12), whereas two patients (no. 8 and 16) carried a previously unreported missense variant (c.1162 T > A) causing p.S388T. A skin biopsy from patient 8 was positive for the c.1162 T > A variant indicating its germ-line origin (data not shown). No mutations were identified in *DNMT3A*, *IDH1* or *IDH2*.

**Table 1:** Clinical characteristics of the MF patients.

Patients	All	
Number	16	
Female	3	19%
Male	13	81%
Age, years (range)	66	(52–80)
Time from diagnosis to inclusion, years median (range)	6.8	(0–22.5)
**Laboratory workup at baseline (median, range)**		
Hemoglobin (g/dL)	10.3	(7.9–13.4)
Leukocytes (x109/L)	5.9	(2.3–64.4)
Platelets (x109/L)	155.5	(56–357)
Lactate dehydrogenase (U/L)	562.5	(195–1841)
Cretinine (micromol/L)	97	(41–185)
Treatment prior to blood sample delivery*		
Hydroxyurea as monotherapy	4	
Hydroxyurea as supplement**	5	
Alpha-interferon	5	
Anagrelide	1	
Darbepoetin alfa***	3	
**Dynamic International Prognostic Scoring System (DIPSS)**		
Low-risk	1	
Intermediate risk 1	9	
Intermediate risk 2	3	
High-risk	2	
Intermediate risk 2 or high-risk****	1	

*Patients had not received any drugs prior to blood sample delivery. **Patients developing ischemic symptoms were given hydroxyurea to control platelet levels. ***A single patient had previously been treated with both darbepoetin alfa and alpha-interferon. ****The blastcount was not known for a single patient meaning that this patient was either in intermediate risk 2 group or scored as a high risk patient.

**Table 2:** Mutational status of *ASXL1*, *TET2*, *JAK2*, *CALR*, and *MPL* in the MF patients.

Patient ID	Chromosomal region (UCSC hg 19)	Gene	Nucleotide change	Amino acid change	Predicted function
1	chr20: 31,022,614	*ASXL1*	c.2099 InsGAG	p.Y700X	Premature termination
1	chr4:106,156,154	*TET2*	c.1118 Del T	p.L392X	Premature termination
1	chr9:5,073,770	*JAK2*	c.1849G > T	p.V617F	Activating mutation
2	chr1:43,815,009	*MPL*	c.1544 G > T	p.W515L	Activating mutation
3	chr20:31,022,592	*ASXL1*	c.2077 C > T	p.R693X	Premature termination
3	chr20:31,022,784	*ASXL1*	c.2269del(25 bp)	Q757fs*6	Frameshift
3	chr19: 13,054,572	*CALR*	c.1099del(52 bp)	p. L367fs*46	Frameshift causing mutant C-terminal
4	chr9:5,073,770	*JAK2*	c.1849G > T	p.V617F	Activating mutation
5	chr20:31,022,983	*ASXL1*	c.2468 T > A	p.L823X	Premature termination
5	chr4:106,158,419	*TET2*	c.3383 C > A	p.S1128X	Premature termination
5	chr9:5,073,770	*JAK2*	c.1849G > T	p.V617F	Activating mutation
6	chr19: 13,054,572	*CALR*	c.1099del(52 bp)	p. L367fs*46	Frameshift causing mutant C-terminal
7	chr20:31,023,472	*ASXL1*	c.2957 A > G	p.N986S	
7	chr9:5,073,770	*JAK2*	c.1849G > T	p.V617F	Activating mutation
8	chr9:5,073,770	*JAK2*	c.1849G > T	p.V617F	Activating mutation
9	chr9:5,073,770	*JAK2*	c.1849G > T	p.V617F	Activating mutation
10	chr20:31,023,717	*ASXL1*	c.3202 C > T	p.R1068X	Premature termination
10	chr1:43,815,009	*MPL*	c.1544 G > T	p.W515L	Activating mutation
11	chr9:5,073,770	*JAK2*	c.1849G > T	p.V617F	Activating mutation
12	chr4:106,155,778	*TET2*	c.742 G > T	p.E248X	Premature termination
12	chr9:5,073,770	*JAK2*	c.1849G > T	p.V617F	Activating mutation
13*	chr9:5,073,770	*JAK2*	c.1849G > T	p.V617F	Activating mutation
14	chr20:31,022,817	*ASXL1*	c.2302 C > T	p.Q768X	Premature termination
14	chr9:5,073,770	*JAK2*	c.1849G > T	p.V617F	Activating mutation
15	chr20:31,022,450	*ASXL1*	c.1935insG	p.G646Wfs*12	Premature termination
15	chr9:5,073,770	*JAK2*	c.1849G > T	p.V617F	Activating mutation
16	chr19: 13,054,572	*CALR*	c.1099del(52 bp)	p. L367fs*46	Frameshift causing mutant C-terminal

*Patient sample 13 was only screened for *JAK2* mutations due to limited material.

Activating mutations of *JAK2* were detected in 11/16 patients (no. 1, 4, 5, 7, 8, 9, 11, 12, 13, 14, and 15) whereas the frameshift *CALR* mutation p.L367fs*46, predicted to cause a C-terminal truncation, was observed in three patients (no. 3, 6, and 16). The activating *MPL* mutation p.W515L was detected in the two patients without *JAK2* and *CALR* mutations (no. 2 and 10).

### Unsupervised cluster analysis did not reveal a genome-wide specific DNA methylation profile associated with *ASXL1* or *TET2* mutations in MF granulocytes or CD34+ cells

**Figure 3:**
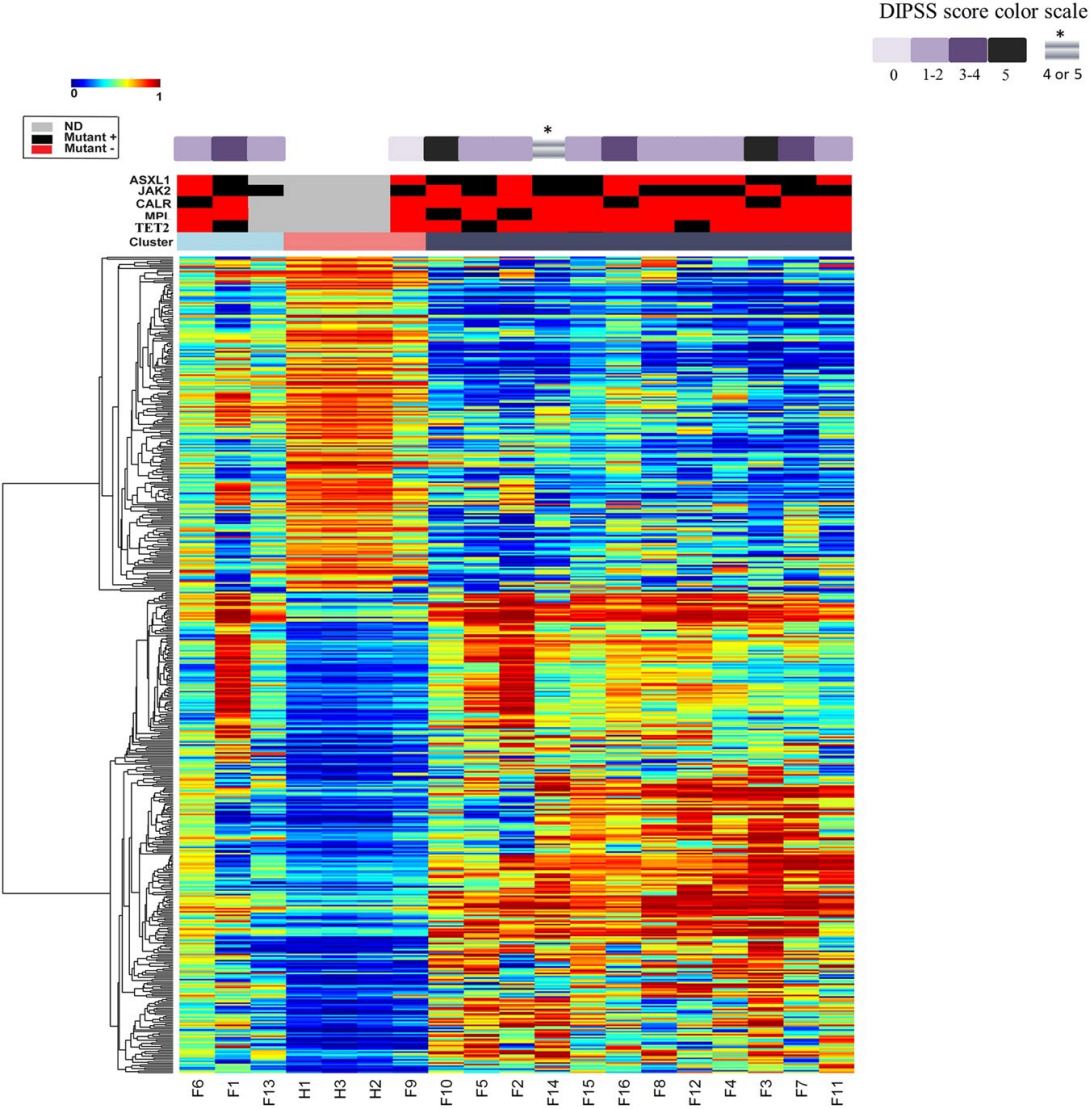
RPMM clustering of the granulocytes and their healthy age-matched counterparts with overlaid mutational status. Fifteen samples were analyzed for mutations in *ASXL1*, *TET2*, *IDH1*, *IDH2*, *DNMT3A*, *CALR*, *JAK2*, and *MPL*, while sample 13 was only analyzed for *JAK2* mutations. The upper purple panel: Dynamic International Prognostic Scoring System (DIPSS). *The blast count was not available for MF patient F14. The middle black and red panel: Mutational status (black represents a mutation). Mutations were found for *ASXL1*, *TET2*, *JAK2*, *CALR*, and *MPL*. Lower panel: Hierarchical clustering of methylation levels in granulocytes from MF patients and healthy age-matched controls. β values range from 0 (blue; unmethylated) to 1 (red; methylated). Columns represent samples and rows represent differentially methylated CpG sites. Euclidean distance and complete linkage were used to study the cluster pattern of differential methylated probes. None of the mutations analyzed were associated with a DNA methylation-based subgrouping.

An unsupervised clustering of granulocytes and CD34+ cells did not identify differential methylation signatures associated with *ASXL1* and *TET2* mutated cases (Figs 3 and S3). RPMM clustering of the 519 CpG sites differentially methylated among MF granulocyte samples and their healthy age-matched controls show three distinct clusters (Fig. 3). Cluster one (light blue) included samples from three patients, whereas cluster two (pink) included the three healthy age-matched controls and a single MF sample (F9). Cluster three (grey) included the remaining 12 MF samples. *ASXL1* mutations were observed in one of two analyzed cases in cluster one and in 50% of patients in cluster 3, indicating that *ASXL1* mutations do not seem to correlate with a specific DNA methylation profile using an unsupervised approach, which is in contrast to a previous study of 12 patients^11^.

### *ASXL1* mutations are associated with differential DNA methylation of tumor suppressors and oncogenes in MF CD34+ cells

**Figure 4:**
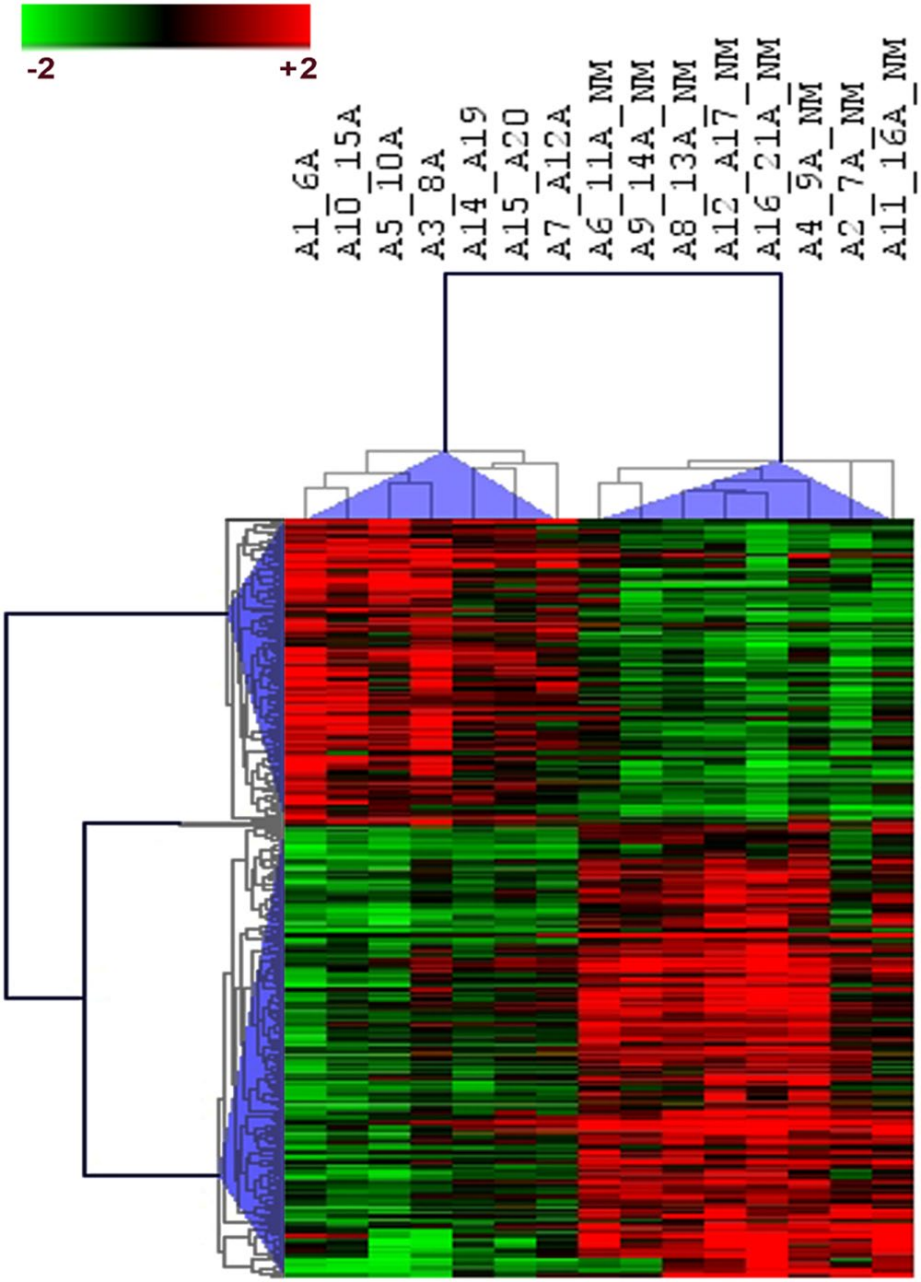
Hierarchical clustering of 308 differentially methylated CpG sites in MF CD34+ cells associated with *ASXL1* mutations using Pearson correlation and Average distance. Green indicates hypomethylated CpG sites and red indicates hypermethylated CpG sites. Columns represent the 15 MF patients analyzed. Rows represent differentially methylated CpG sites in MF CD34+ cells in *ASXL1* mutated (n = 7) and *ASXL1* non-mutated (_NM) (n = 8) cases.

We next performed a supervised cluster analysis to investigate the association between mutated *ASXL1* cases and aberrant DNA methylation in MF CD34+ cells. We identified 308 differentially methylated CpG sites (with FDR p < 0.05; |Δβ| ± 0.2) annotated to 174 genes (Table S5) associated with mutated *ASXL1*, which we named the “*ASXL1* methylation signature” (Fig. 4). Of the 308 CpG sites 124 were hypermethylated while 184 were hypomethylated. In the granulocyte population a supervised cluster analysis identified 281 differentially methylated CpG sites (with FDR p < 0.05; |Δβ| ± 0.3) annotated to 137 genes (Table S6) associated with mutated *ASXL1*. Of the 281 CpG sites 105 were hypermethylated while 176 were hypomethylated. Several tumor suppressors and oncogenes including *RASSF1*, *miR-663*, *ARID5B*, *FIP1L1*, *BCL6*, *TRPM2*, *ADORA1*, *ADORA2A*, *TFA2PA*, and *DIRC2* were differentially methylated in the *ASXL1* mutated cases (Table S7).

### *ASXL1* mutations correlate with differential methylation in CpG rich regions in MF CD34+ cells

**Figure 5:**
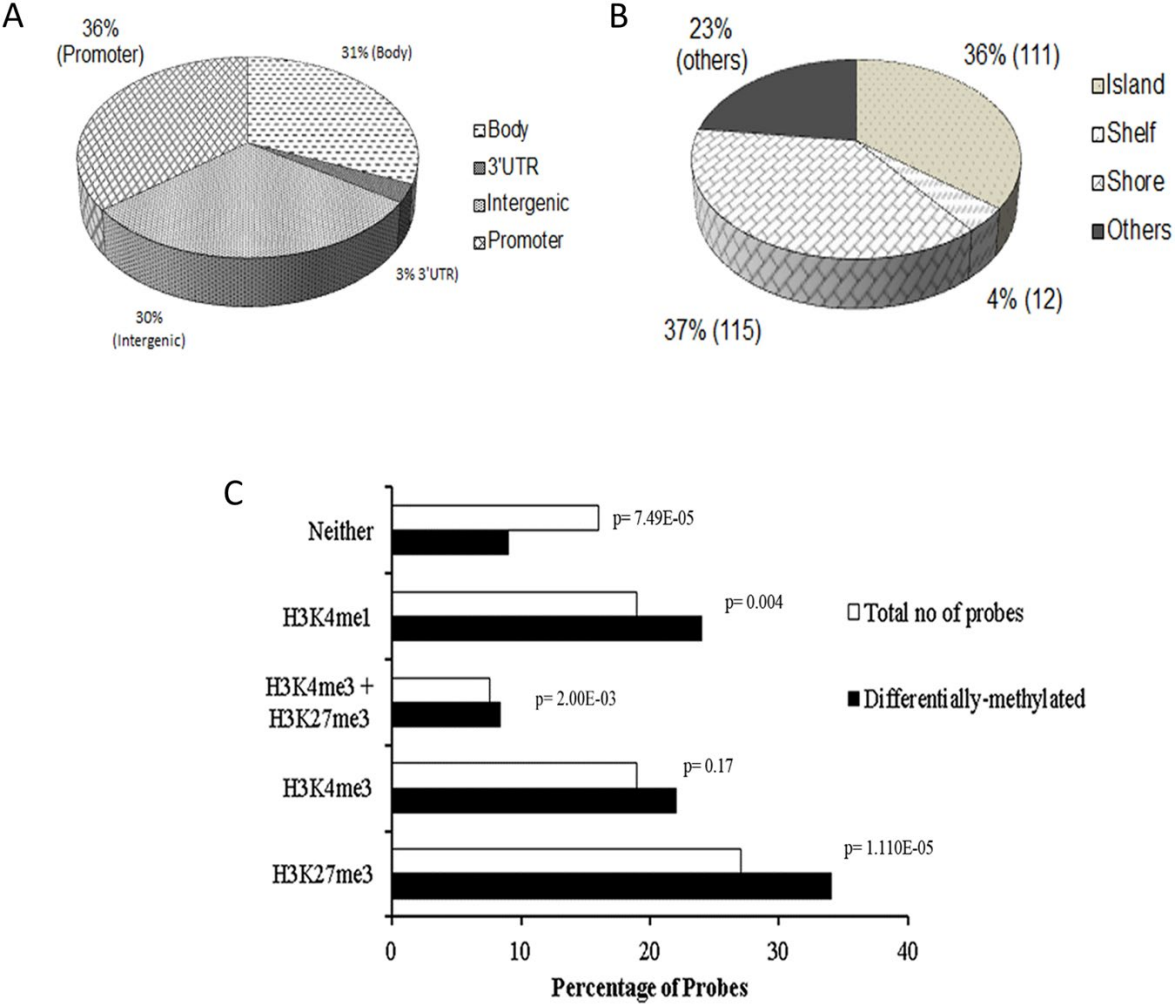
The relative distribution of the differentially methylated CpG sites associated with *ASXL1* mutations in CD34 + MF cells. (**A**) Functional genomic distribution in gene body, 3’UTR, intergenic, and promoter; and (**B**) mapping according to the CpG density to islands, shelf, shore, and others/open sea. The majority of CpG sites were mapped to CpG shores (37%) and CpG islands (36%). (**C**) *ASXL1* associated differentially methylated CpG sites were enriched in regions enriched for the histone marks H3K4me1 (P = 0.004), H3K27me3 (P = 1.10E-05), and the bivalent mark H3K27me3 plus H3K4me3 (P = 2.00E-03) in healthy CD34+ cells.

We mapped the 308 “*ASXL1* methylation signature” CpG sites according to genomic regions. In samples with *ASXL1* mutations, 36% of the differentially methylated CpG sites mapped to promoter regions, 31% to gene bodies, 30% to intergenic regions, and 3% to 3′UTRs (Fig. 5A). The majority of these regions were CG rich with 36% of the affected areas categorized as CpG islands and 37% as CpG shores (Fig. 5B).

### *ASXL1* methylation signature probes are enriched in regions that carry H3K27me3, H3K4me1, and H3K27me3 plus H3K4me3 in CD34+ cells

Regions enriched with the repressive mark H3K27me3 and the bivalent histone mark H3K27me3 plus H3K4me3 were found to have a significantly higher number of differentially methylated CpG sites in *ASXL1* mutated cases (Fig. 5C). Both hypo- and hypermethylation of CpG sites were observed in regions enriched for H3K27me3 and H3K27me3 plus H3K4me3 histone marks (Table S5). In addition, regions enriched with H3K4me1 in CD34+ cells were also found to have a significant higher number of differentially methylated CpG sites in patients with *ASXL1* mutations compared to non-mutated *ASXL1* cases (Fig. 5C) of which the majority of the CpG sites (93/120) were hypomethylated (Table S5).

## Discussion

As MF involves both hematopoietic progenitors and more mature cells, a broad spectrum of cells throughout the myeloid compartment may be affected, but the contribution of the individual cell types to MF pathogenesis has not previously been explored. A previous study has shown correlation of *ASXL1* mutations to a higher overall DNA methylation level and leukemic transformation in MF, whereas *TET2* mutations correlated with increased DNA methylation levels of a distinct set of genes^11^, however, that study was based on the analyses of only 12 cases and needs confirmation in a larger cohort.

In our study, DNA methylation profiling of sorted MF cells and normal counterparts revealed that all three cell populations studied were characterized by distinct differential DNA methylation patterns. Interestingly, we observed that the majority of differentially methylated CpG sites were only differentially methylated in particular cellular compartments. This is likely indicative that, within each individual cell type, different DNA methylation patterns have a specific contribution to MF pathogenesis, rather than just being associated with lineage. To validate the genome-wide DNA methylation data a set of 4 genes (*LEP*, *TRIM59*, *ZNF577*, and *WT1*), that were found hypermethylated in the CD34 + compartment, were analyzed in a validation cohort of 30 MF patients where DNA had been isolated from whole blood. The fact that hypermethylation was confirmed in the validation cohort underline the presence of a MF specific methylation pattern, and opens up the potential of DNA methylation-based biomarkers for clinical purposes. Of the four genes analyzed in our validation cohort *WT1* is especially interesting as it is found upregulated in myelofibrosis^23^, which corresponds to our finding of increased gene body methylation. WT1 has been shown to contribute to the plasticity of DNA methylation by recruiting TET2 to target genes causing site-specific demethylation^24^. A functional role of the remaining three genes *LEP*, *TRIM59*, and *ZNF577* in MF pathogenesis still needs to be established and will require functional studies.

The MF CD34+ population had most differentially methylated CpG sites and, according to the pathway analyses, the genes with differentially methylated CpG sites were involved in ‘hematopoietic differentiation’, ‘cell-cycle’, ‘cell death and survival’, and ‘cancer’, probably contributing to the increased proliferation and dedifferentiation observed in these cells. The mononuclear cells had the lowest number of differentially methylated CpG sites, but interestingly, differentially methylated genes were associated with ‘immunological disease’, ‘cell death and survival’, and ‘cancer’. These aberrations may at least to some extent be linked to the high level of inflammation observed in MPN^25, 26^. Further sorting of the mononuclear cells could potentially reveal a more profound understanding of the contribution from the different subtypes. Genes that were found differentially methylated in the granulocytes were involved in ‘inflammatory disease’, ‘cell cycle’, ‘hematological disease’, and ‘cancer’. These data imply that specific characteristics of the malignant clones in the individual cellular compartments may contribute to different aspects of the MF phenotype.

Since DNA methylation has been associated with mutations in epigenetic regulators we next analyzed the mutational status of epigenetic regulators in our MF cohort. Sequencing analyses showed that *ASXL1* was mutated in 7/15 (47%) patients, where a premature stop codon predicted to result in truncation in six cases, indicating that normal ASXL1 function may be lost. *TET2* mutations were observed in 3/15 (20%). Thus, the frequency of mutations observed in our cohort is consistent with that of others, who report *ASXL1* mutations in 20–55%^14, 27, 28^, and *TET2* mutations in 14–20%^29, 30^. Mutations of *IDH1*, *IDH2* and *DNMT3A* in MF are infrequent ranging from 0–7%^14, 29–32^.

With 7/15 patients having *ASXL1* mutations, we aimed to investigate the *ASXL1* mutation associated DNA methylation signature in MF. In contrast to a previous study of 12 patients^11^, we did not find *ASXL1* mutations to be associated with an overall higher level of DNA methylation, or a distinct DNA methylation profile in either the granulocytes or the CD34+ cells using an unsupervised approach. However, when using supervised clustering analysis in the CD34+ cells a subset of differentially methylated CpG sites, frequently located in tumor suppressors and oncogenes, were found in *ASXL1* mutated cases.

The majority of differentially methylated CpG sites associated with the “*ASXL1* methylation signature” in CD34+ cells were enriched in regions with the repressive histone mark H3K27me3, whereas a minor proportion of the differentially methylated CpG sites were enriched in regions with the bivalent histone mark H3K27me3 plus H3K4me3. This is remarkable because ASXL1 has been suggested to regulate histone H3K27 methylation through interactions with the Polycomb-repressive complex 2 (PRC2). However, the association between *ASXL1* mutation, H3K27me3, and differential DNA methylation is not straight forward and warrants further study. Differentially methylated CpG sites in *ASXL1* mutated cases were also found in regions with the active histone mark H3K4me1, found at enhancer regions, which is likely to influence transcription of nearby genes. Most of the CpG sites overlapping with H3K4me1 were hypomethylated, and may thus possibly be associated with enhancer activation. Several genes previously recognized as tumor suppressors and oncogenes in other cancers including e.g. *RASSF1*, *miR-663*, *ARID5B*, *FIP1L1*, *BCL6*, *TRPM2*, *ADORA1*, *ADORA2A*, *TFA2PA*, and *DIRC2* were among the “*ASXL1* methylation signature genes”. Our data indicate that truncated ASXL1 is associated with methylation changes of a distinct set of cancer related genes that may be involved in disease progression, although no direct link between ASXL1 and DNA methylation has yet been established. Extending these analyses to *TET2* mutated cases had been interesting but with only three MF patients carrying a *TET2* mutation, of which two also had an *ASXL1* mutation, we decided to focus on *ASXL1* only. In addition, it would have been interesting to extent these analyses to *JAK2* as JAK2 has been shown to influence the chromatin directly by phosphorylation of histone H3 tyrosine 41^33^. Indirectly, through the phosphorylation of PRMT5, mutated *JAK2V617F* has been shown to result in reduced methylation at histone H2A or H4 at R3^34^. A direct link between JAK2 and DNA methylation is, however, missing and previous studies have not shown any association between *JAK2* mutations and DNA methylation in myelofibrosis^11, 12^.

Taken together we found that aberrant and variable methylation patterns are present in the different myeloid cell compartments of MF patients. The differentially methylated CpG sites are annotated to several tumor suppressor genes and oncogenes, but also to genes involved mainly in inflammation and immunological diseases. Thus, the MF phenotype is likely a result of the aberrant function of distinct cell types throughout the myeloid lineages. In addition, we found that *ASXL1* mutations are associated with DNA methylation changes in regulatory regions of cancer associated genes, not previously associated with MF. In future studies it shall be interesting to explore if there is a direct link between *ASXL1* mediated gene regulation and aberrant methylation of these genes in malignant myelopoiesis.

## Methods

### Patient material and controls

This study is based on a primary cohort of 16 MF patients and three healthy age-matched controls and a validation cohort of 30 MF patients and 11 healthy controls. Clinical characteristics of the primary MF cohort including the Dynamic International Prognostic Scoring System (DIPSS) are shown in Table 1. For the primary cohort peripheral blood was separated into granulocytes and mononuclear cells using a Ficoll gradient. As a consequence of fibrotic bone marrow and extramedullary hematopoiesis, MF CD34+ cells could be isolated from the fraction of mononuclear cells using a CD34 + positive selection kit on a RoboSep^TM^ platform (Stemcell Technologies, Grenoble, France). CD34+ cells from bone marrow, peripheral blood granulocytes, and mononuclear cells from three healthy age-matched individuals were used as controls. DNA was extracted using the AllPrep DNA/RNA Mini Kit (Qiagen, Hilden, Germany).

DNA from whole blood was extracted from the validation cohort using the Autopure LS (Qiagen) instrument and the Gentra Puregene Blood Kit (Qiagen), respectively.

The study was approved by the regional ethical committee (De Videnskabsetiske Komitéer Region Hovedstaden, Journal: H-C-2008–079) and all experiments were performed in accordance with the approved guidelines and regulations. All patients included had given a written informed consent.

### Genome-wide DNA methylation profiling

Genome-wide DNA methylation profiling was performed using the 450 K Infinium array (Illumina Inc, San Diego, USA) platform as desribed previously^35^. This platform interrogates the methylation status of more than 480,000 CpGs in the human genome corresponding to 99% of NCBI RefSeq genes, which include CpGs in the promoters, enhancers, and gene bodies among others. In addition, the array covers CpG islands, shores and shelves of CpG islands. After hybridization and scanning of BeadChips, IDAT files were extracted to calculate the DNA methylation score (β values) ranging from 0 (non-methylated) to 1 (fully methylated) as described previously^35^.

Data filtering and normalization of DNA methylation data: Measurements in which the fluorescence intensity was not statistically significant above background signal were removed from the data set. Through an initial filtering process, probes corresponding to X and Y chromosomes and those containing a single nucleotide polymorphism (SNP) within five base pairs of targeted CpG sites were excluded. Probes with a repetitive element in the probe sequence within five bases of the targeted CpG site were also excluded. In total 361974 probes were used for further analysis.

Differential methylation between the MF samples and healthy control samples of individual CpG sites for each cell type was calculated. The probes with a FDR p < 0.05 in t-test and Δβ >  ± 0.2 were considered to be differentially methylated as previously described^35^, with the exception of the granulocytes for which a mean Δβ >  ± 0.3 was used with an adjusted p value < 0.01. For visualization, RPMM (recursively partitioned mixture model) and hierarchical clustering approaches were used. Hierarchial clustering using Euclidean distance and average linkage was used to classify samples into various groups as described previously^36^. All statistical analyses and clustering were performed using a R-statistical packages (https://www.r-project.org/) as described previously^35, 37^.

### Pathway analysis

Functional interpretation of genes with one or more significantly differentially methylated CpG sites annotated was analyzed in the context of gene ontology and molecular networks by using Ingenuity pathway software (IPA; www.ingenuity.com) as described previously^35^.

### Enrichment analysis

To investigate whether differentially methylated CpG sites were enriched in regions with distinct histone modifications, including H3K4me1, H3K4Me3, H3K4me3 plus H3K27me3, and H3K27me3, ChIP-seq data from human CD34+ cells was downloaded from NIH roadmap Epigenomics mapping consortium (http://www.roadmapepigenomics.org/) and GSE36994, and the coordinates of ChIP-seq peaks were mapped to the 450 K probe locations. A hypergeometric test was used to evaluate possible enrichment of differentially methylated CpG sites in regions with distinct histone modifications.

### Validation of differentially methylated sites using pyrosequencing

Four genes (*LEP*, *TRIM59*, *WT1*, and *ZNF577*) with at least two differentially methylated CpG sites in close proximity to the transcription start site (Table S1) were further analyzed in whole blood from a validation cohort of 30 MF patients and 11 healthy controls. Methylation independent (MIP) assays^38^ were designed using the PyroMark Assay Design 2.0 (Qiagen). The PCR amplicons were pyrosequenced on the PyroMark Q24 (Qiagen) instrument using the PyroMark Gold Q24 reagents (Qiagen) according to manufacturers’ instructions. For each of the four genes the DNA methylation level is calculated as the median DNA methylation level of the CpG sites included in the assay (Table S1). Primer sequences and PCR conditions are given in Table S1.

### Mutation analysis

Mutation analyses were performed on DNA extracted from MF granulocytes. The primer sequences and assay conditions for the mutation analyses of the genes of interest have previously been published; *TET2*, *IDH1*, *IDH2*, *DNMT3A*^35^, *JAK2*^39^, *CALR*^7^, and *MPL*^40^. *ASXL1* exon 12 was analyzed for mutations as previously described^15^ with modifications for two assays (Table S1). M13 tagged primers were used for *CALR*, *ASXL*, and *MPL*.

## Electronic supplementary material

Supplementary Dataset 1
