# Publisher Correction: Epigenetic changes in myelofibrosis: Distinct methylation changes in the myeloid compartments and in cases with *ASXL1* mutations

**DOI:** 10.1038/s41598-018-35596-w

**Published:** 2018-11-20

**Authors:** Helene Myrtue Nielsen, Christen Lykkegaard Andersen, Maj Westman, Lasse Sommer Kristensen, Fazila Asmar, Torben Arvid Kruse, Mads Thomassen, Thomas Stauffer Larsen, Vibe Skov, Lise Lotte Hansen, Ole Weis Bjerrum, Hans Carl Hasselbalch, Vasu Punj, Kirsten Grønbæk

**Affiliations:** 1 Department of Hematology, Rigshospitalet, Copenhagen University Hospital, Copenhagen, Denmark; 2 Department of Biomedicine, Aarhus University, Aarhus, Denmark; 3 Danish Stem Cell Centre (DanStem) Faculty of Health Sciences, University of Copenhagen, Copenhagen, Denmark; 4 Department of Hematology, Roskilde Hospital, Roskilde, Denmark; 5 Department of Clinical Genetics, Rigshospitalet, Copenhagen, Denmark; 6 Department of Clinical Genetics, Odense University Hospital, Odense, Denmark; 7 Department of Hematology, Odense University Hospital, Odense, Denmark; 8 Division of Hematology, Keck School of Medicine, University of Southern California, Los Angeles, United States

Correction to: *Scientific Reports* 10.1038/s41598-017-07057-3, published online 28 July 2017

In the original version of this Article, the authors Helene Myrtue Nielsen, Christen Lykkegaard Andersen, Lasse Sommer Kristensen, Torben Arvid Kruse, Thomas Stauffer Larsen, Lise Lotte Hansen, Ole Weis Bjerrum and Hans Carl Hasselbalch were incorrectly indexed. This error has now been corrected.

