# Supplementary material for: Epigenetic changes in myelofibrosis: Distinct methylation changes in the myeloid compartments and in cases with ASXL1 mutations
Source: Sci Rep. 2017 Jul 28;7:6774. doi: 10.1038/s41598-017-07057-3 (PMC5533802; doi:10.1038/s41598-017-07057-3)
Supplement: Supplementary file 1 — Supplementary Dataset 1 [file 41598_2017_7057_MOESM1_ESM.pdf]

Supplementary

Epigenetic changes in myelofibrosis: Distinct methylation changes in the myeloid compartments and in cases with *ASXL1* mutations

Helene Myrtue Nielsen[1, 2, 3], Christen Lykkegaard Andersen[1, 4], Maj Westman[5], Lasse Sommer Kristensen[1], Fazila Asmar[1], Torben Arvid Kruse[6], Mads Thomassen[6], Thomas Stauffer Larsen[7], Vibe Skov[4], Lise Lotte Hansen[2], Ole Weis Bjerrum[1], Hans Carl Hasselbalch[4], Vasu Punj[8], and Kirsten Grønbæk[1, 3]

[1]Department of Hematology, Rigshospitalet, Copenhagen University Hospital, Copenhagen, Denmark
[2]Department of Biomedicine, Aarhus University, Aarhus, Denmark
[3]Danish Stem Cell Centre (DanStem) Faculty of Health Sciences, University of Copenhagen, Copenhagen, Denmark
[4]Department of Hematology, Roskilde Hospital, Roskilde, Denmark
[5]Department of Clinical Genetics, Rigshospitalet, Copenhagen, Denmark
[6]Department of Clinical Genetics, Odense University Hospital, Odense, Denmark
[7]Department of Hematology, Odense University Hospital, Odense, Denmark
[8]Keck School of Medicine, University of Southern California, Los Angeles, United States

Supplementary Figure Legends

Supplementary Figure 1. Hierarchical Clustering using Euclidean distance and average linkage of 504 probes with SD > 0.30 across all samples. The samples are designated as: MF CD34+ cells as A, healthy CD34+ cells as C, MF mononuclear cells as D, healthy mononuclear cells as E, MF granulocytes as F, and healthy granulocytes as H. The granulocytes are characterized by a lower overall DNA methylation level. A single MF granulocyte sample (F16) had a higher overall DNA methylation level compared to the remaining granulocyte samples and therefore clustered with the CD34+ and mononuclear cells.

Supplementary Figure 2. Functional gene ontology for differentially methylated genes in the CD34+ cell, mononuclear cells, and granulocytes respectively. The pathways affected by differential methylation are ranked by their P value (Y-axis) for the CD34+ cells (2A), the mononuclear cells (2B), and for the granulocytes (2C).

Supplementary Figure 3. RPMM clustering of the CD34+ MF cells and their healthy age-matched counterparts with overlaid mutational status. Fifteen samples were analyzed for mutations in *ASXL1*, *TET2*, *IDH1*, *IDH2*, *DNMT3A*, *CALR*, *JAK2*, and *MPL*, while sample 13 was only analyzed for *JAK2* mutations.

The upper panel: Mutational status (black represents a mutation). Mutations were found for *ASXL1*, *TET2*, *JAK2*, *CALR*, and *MPL*.

Lower panel: Hierarchical clustering of methylation levels in the CD34+ cells from MF patients and healthy age-matched controls. β values range from 0 (blue; unmethylated) to 1 (red;

methylated). Columns represent samples and rows represent differentially methylated CpG sites. Euclidean distance and complete linkage were used to study the cluster pattern of differential methylated probes. None of the mutations analyzed were associated with a DNA methylation-based subgrouping.

Supplementary Figures

Supplementary Figure 1

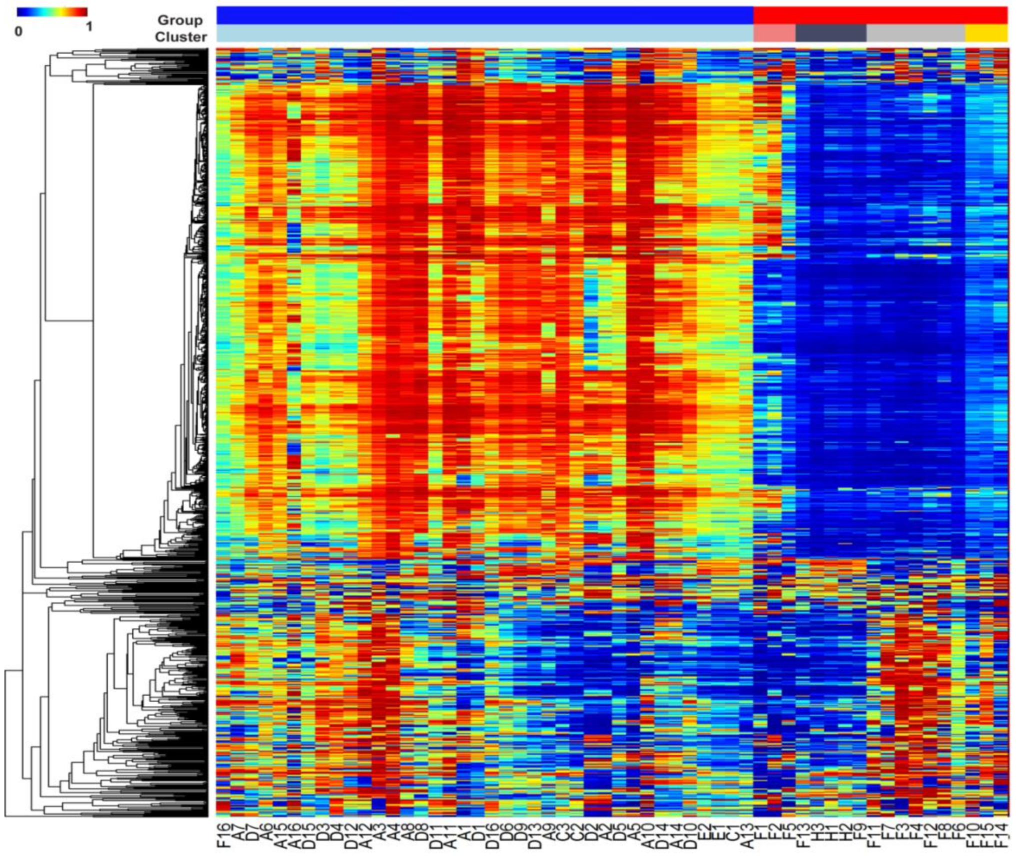

Supplementary Figure 2

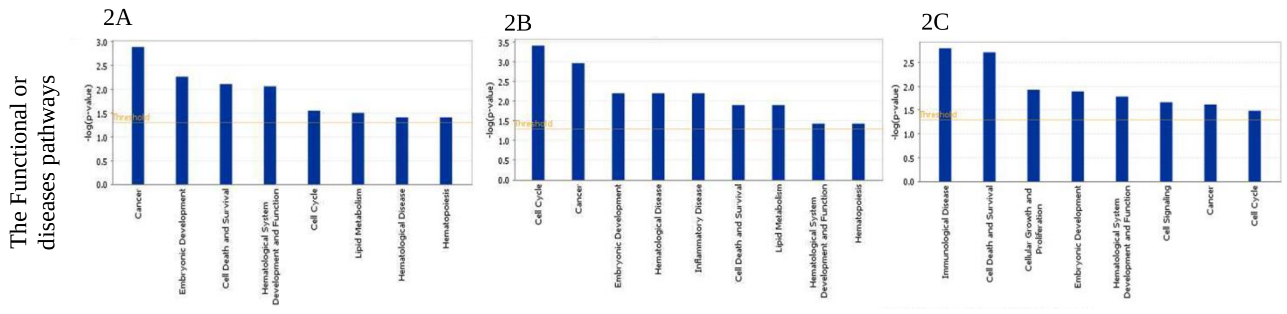

Supplementary Figure 3

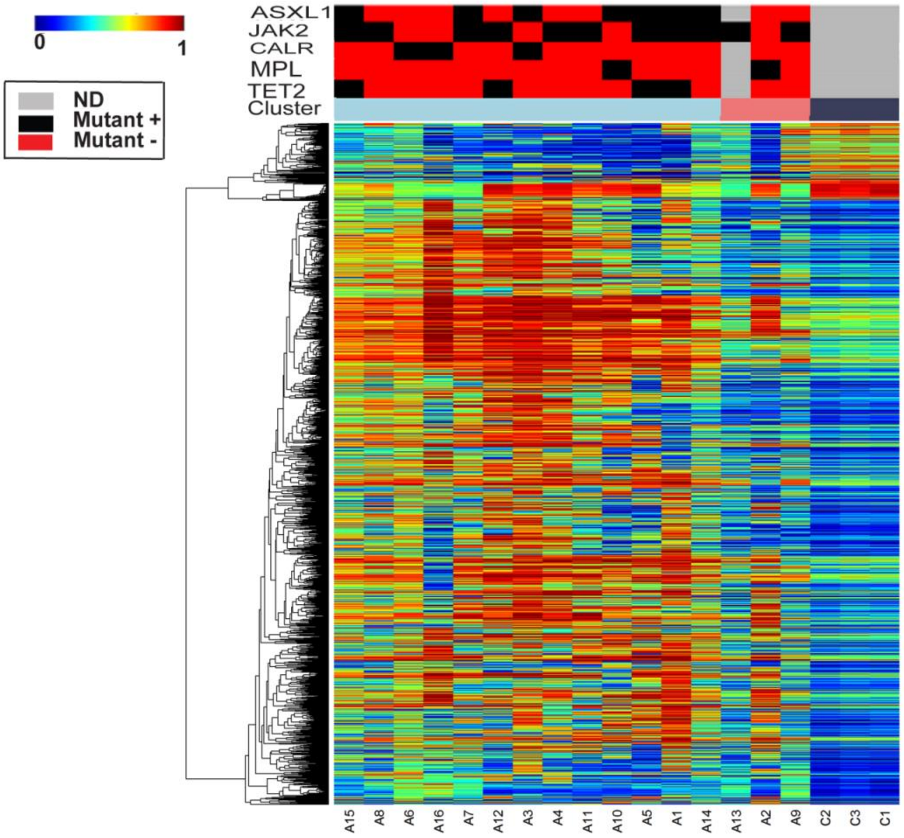

Supplementary Tables

Supplementary Table 1. Primer sequences for the DNA methylation and mutation analyses

Gene name	Primer sequence	Region amplified (UCSC hg 19)	Number of CpG sites analyzed	Ref.
**DNA methylation analysis***				
LEP	F: 5’-ggggTagttgagTaagttgtgat<br>R: 5’-Bio-AaactaaAccatcctccctAcatccct<br>Seq: 5’-gggTaggTatggagT	Chr7:127,881,270-127,881,484	4	**
TRIM59	F: 5’-gatgtgggtgttTTTtgagaat<br>R: 5’-Bio-caAtAAAcctcctAaActAAAAtccc<br>Seq: 5’-tgtggagTTaagtaaaagataattT	Chr3:160,167,883-160,168,132	8	**
ZNF577	F: 5’-ggaTtggaaaaagggtgaaatg<br>R: 5’-Bio-aaccccacacactAcaAac<br>Seq: 5’-aaagggaaggTtggtg	Chr19:52,391,168-52,391,437	4	**
WT1	F: 5’-gagaatTtgggatTaTtgtTTtgtgga<br>R: 5’-Bio-tccccaaaaAtaacaccaAcctAc<br>Seq: 5’-agtgaaattagtgttttgtaT	Chr11:32,454,681-32,454,873	2	**
**Mutation analysis**				
**Gene name**	**Primer sequence**	**Region amplified (UCSC hg 19)**	**Amplicons size (bp)**	**Ref.**
ASXL1 Exon12	F1: 5’-AGGTCAGATCACCCAGTCAGTT<br>R1: 5’-TAGCCCATCTGTGAGTCCAACTGT	Chr20:31,022,184-31,022,744	561	[1]
	F2: 5’-GAGAGGACCTGCCTTCTCTG<br>R2: 5’-TGGGCTGTTTCACTACCTCA	Chr20:31,022,668-31,023,249	582	***
	F3: 5’-ACTTGAAAACCAAGGCTCTCGT<br>R3: 5’-GCAACCATCCCATCTGTCCTTGTA	Chr20:31,023,166-31,023,697	532	[1]
	F4: 5’-GGTGGACAAGGATGAGAAACCCAA<br>R4: 5’-TGTCCTGTGACATAGCACGGACTT	Chr20:31,023,599-31,024,272	674	[1]
	F5: 5’-TCAGAAGGAAGTCCGTGCTA<br>R5: 5’-AGTTGGGAGGGGAGAGAAGA	Chr20:31,024,241-31,024,816	576	***
	F6: 5’-ACAGGAAAGCTACTGGGCATAGTC<br>R6: 5’-CAAGAGTGCTCCTGCCTAAAGAGT	Chr20:31,024,666-31,025,258	593	[1]

* Colored letters are designed to be a mismatch to cytosines followed by guanine to avoid PCR bias.
** Assays are designed using the PyroMark Assay Design software from Illumina.
***Assays are designed in house using the software Primer 3.

Supplementary Table 2. The 200 most significantly differentially probes between MF CD34+ cells and their healthy age-matched controls. Red indicates hypomethylation and green indicates hypermethylation.

Illumids	Chromo-some	Cytosine position at the chromosome	No of probes annotated to the gene	Gene name	Regulatory site annotated
cg02254774	11	50257496	1	LOC441601	Body
cg19087971	7	751233	3	PRKAR1B;PRKAR1B;PRKAR1B;PRKAR1B;PRKAR1B;PRKAR1B	5'UTR;5'UTR;5'UTR;5'UTR;5'UTR;5'UTR
cg16592832	22	24891141	1	C22orf45;C22orf45;UPB1	TSS1500;TSS1500;TSS200
cg23010048	19	52391257	10	ZNF577;ZNF577;ZNF577	TSS200;TSS200;TSS200
cg13170076	2	382356			
cg02336143	5	140769170	43	PCDHGB4;PCDHGA4;PCDHGA6;PCDHGA1;PCDHGA5;PCDHGB1;PCDHGA3;PCDHGA2;PCDHGA7;PCDHGB2;PCDHGB4;PCDHGB3	1stExon;Body;Body;Body;Body;Body;Body;Body;Body;Body;1stExon;Body
cg02161292	5	140821687	43	PCDHGA4;PCDHGA12;PCDHGA11;PCDHGA11;PCDHGA9;PCDHGA1;PCDHGB1;PCDHGB6;PCDHGB3;PCDHGB7;PCDHGA6;PCDHGA8;PCDHGA10;PCDHGA5;PCDHGB4;PCDHGA3;PCDHGA2;PCDHGB2;PCDHGA7;PCDHGB5	Body;Body;Body;Body;Body;Body;Body;Body;Body;Body;Body;Body;Body;Body;Body;Body;Body;Body;Body;Body
cg21461300	13	114830702	1	RASA3	Body
cg23350350	8	16652008			
cg20553766	20	54824583	1	MC3R	1stExon
cg12635919	1	3319488	8	PRDM16;PRDM16	Body;Body
cg05168491	14	38080446			
cg08027484	6	10556523	1	GCNT2;GCNT2;GCNT2	5'UTR;1stExon;Body
cg01936370	3	156838174			
cg05890377	2	74357713			
cg18394552	5	159428643			
cg01862688	5	140480872	1	PCDHB3	1stExon
cg27436286	15	65670863	1	IGDCC3	TSS1500
cg07586026	1	12600225			
cg09122035	11	319667			
cg02254574	17	32483640	3	ACCN1;ACCN1	1stExon;5'UTR
cg08173263	19	14276911	1	LPHN1;LPHN1	Body;Body
cg02772995	2	161348781	2	RBMS1;RBMS1	Body;Body
cg02838492	9	116861288	1	KIF12;KIF12	1stExon;5'UTR
cg21012125	1	14838453			
cg15618978	3	160167990	2	TRIM59	TSS1500
cg00003578	1	12600529			
cg16731240	19	52391250	10	ZNF577;ZNF577;ZNF577	TSS200;TSS200;TSS200
cg26389950	5	140562236	1	PCDHB16	1stExon
cg25447806	3	156838096			
cg21715903	6	51275527			
cg08255481	16	88103035	1	BANP;BANP (= SMAR1 and SMARBP1)	Body;Body

cg25288068	6	28793681			
cg13625026	11	1146419			
cg06792262	3	44622596	1	ZNF167	Body
cg11269599	19	52391304	10	ZNF577;ZNF577;ZNF577	TSS200;TSS200;TSS200
cg10635122	19	52391090	10	ZNF577;ZNF577;ZNF577;ZNF577	Body;5'UTR;5'UTR;1stExon
cg03060468	5	140683196	3	SLC25A2	1stExon
cg19037350	5	140734648	43	PCDHGA4;PCDHGA4;PCDHGA2;PCDHGA1;PCDHGB1;PCDHGA3	TSS200;TSS200;Body;Body;Body;Body
cg08231710	1	1566687	1	MMP23A;MMP23B	TSS1500;TSS1500
cg15701612	11	130253770			
cg12968518	2	148602504	1	ACVR2A	TSS200
cg17280346	3	147126703	11	ZIC1	TSS1500
cg08451957	2	114256406	3	FOXD4L1	TSS1500
cg14862806	17	21356311			
cg24082121	5	672872	2	TPPP	Body
cg17438030	5	72526379			
cg05609536	19	41019285	1	SPTBN4	Body
cg21882300	17	64831087			
cg15257565	10	42644887			
cg08854834	21	30517941	1	C21orf7	Body
cg10530889	10	93805590			
cg18062721	3	11643427	1	VGLL4;VGLL4	Body;Body
cg20902757	8	67454892			
cg24471254	7	100253792	1	ACTL6B	Body
cg06878361	19	52390810	10	ZNF577;ZNF577;ZNF577	Body;5'UTR;5'UTR
cg08818610	6	24910720	9	FAM65B	5'UTR
cg08164151	12	131118432			
cg01557951	1	50892299			
cg03134157	8	145926089			
cg05772104	2	98340425	1	ZAP70	5'UTR
cg18566727	14	19614829			
cg09141303	4	103998291	2	NHEDC2	TSS200
cg12243267	19	57630536	1	USP29	TSS1500
cg19092396	1	1143751			
cg21572722	6	11044894	3	ELOVL2	TSS1500
cg15915602	2	114737459			
cg10668414	20	34895979			
cg20488281	12	119772354	3	CCDC60	TSS200
cg03562414	19	52391078	10	ZNF577;ZNF577;ZNF577	Body;5'UTR;5'UTR
cg17179314	15	78933820	1	CHRNB4	TSS1500
cg06819935	16	50003431			
cg05618934	4	1407592			
cg16647921	4	41867533			
cg24950222	19	43969886	4	LYPD3 (=C4.4A)	TSS200
cg14646039	5	152729842			
cg00806481	1	2996650	8	PRDM16;PRDM16	Body;Body
cg11101926	17	32483711	3	ACCN1;ACCN1	1stExon;5'UTR
cg08352755	5	145758879			
cg18806707	7	37998416			
cg12424921	11	63828039	2	MACROD1	Body
cg05491854	6	24910562	9	FAM65B	5'UTR
cg26365545	5	76932016	4	OTP	Body
cg24843346	8	142736091			
cg22879098	5	672845	2	TPPP	Body
cg02637101	15	99548944	1	LOC145814;LOC145814	5'UTR;TSS200
cg19431274	5	72714744			
cg05472062	10	93559124	1	TNKS2	Body
cg12309360	5	177412636			
cg04259907	19	43969884	4	LYPD3	TSS200
cg13245431	22	30476525	1	HORMAD2	5'UTR

cg20468415	8	41592973	1	ANK1;ANK1;ANK1;ANK1;ANK1	Body;Body;Body;Body;Body
cg06530441	22	51016950	3	CPT1B;CPT1B;CPT1B;CHKB-CPT1B;CPT1B;CPT1B;CPT1B;CPT1B	TSS200;TSS1500;5'UTR;Body;5'UTR;5'UTR;5'UTR;TSS200
cg03497399	3	33260934	1	SUSD5	TSS1500
cg01224715	5	140811520	43	PCDHGA4;PCDHGA11;PCDHGA11;PCDHGA9;PCDHGA1;PCDHGB1;PCDHGB6;PCDHGA12;PCDHGB3;PCDHGB7;PCDHGA6;PCDHGA8;PCDHGA10;PCDHGA12;PCDHGA5;PCDHGB4;PCDHGA3;PCDHGA2;PCDHGA7;PCDHGB2;PCDHGB5	Body;Body;Body;Body;Body;Body;Body;1stExon;Body;Body;Body;Body;Body;1stExon;Body;Body;Body;Body;Body;Body;Body
cg00946921	14	106025021			
cg06991392	1	147789842			
cg09550809	5	78407562	5	BHMT	TSS200
cg25126698	5	140751840	43	PCDHGA4;PCDHGB3;PCDHGA1;PCDHGA5;PCDHGB1;PCDHGA3;PCDHGB3;PCDHGA2;PCDHGB2	Body;1stExon;Body;Body;Body;Body;1stExon;Body;Body
cg13658899	9	139094665	1	LHX3;LHX3	Body;Body
cg08190125	14	106145435			
cg11439821	15	48483834	1	CTXN2	TSS200
cg17277199	2	24397845	1	C2orf84	TSS200
cg18019451	1	151746047	1	TDRKH;TDRKH;TDRKH;TDRKH	3'UTR;3'UTR;3'UTR;3'UTR
cg23580000	16	50322156	1	ADCY7	1stExon
cg26814075	7	127881298	4	LEP	TSS200
cg18700428	16	3243304			
cg00513288	19	2358394			
cg07330212	5	140202922	12	PCDHA2;PCDHA1;PCDHA1;PCDHA3;PCDHA4;PCDHA5;PCDHA5	Body;Body;Body;Body;Body;1stExon;1stExon
cg24566261	6	30854164	1	DDR1;DDR1	5'UTR;5'UTR
cg19997861	6	31526079	1	NFKBIL1;NFKBIL1;NFKBIL1;NFKBIL1	Body;Body;Body;Body
cg12750917	1	198204074	1	NEK7	Body
cg13381984	7	127881344	4	LEP;LEP	1stExon;5'UTR
cg01963623	22	18632433	3	USP18	TSS1500
cg01810575	17	79415682	1	BAHCC1	Body
cg07419021	2	162274436	1	TBR1	Body
cg16363146	11	22689281	1	GAS2;GAS2	TSS1500;5'UTR
cg11762968	13	95354190			
cg20376009	16	34296108			
cg16221240	2	130970934			
cg06176824	16	33039897			
cg13836550	10	118084504	1	C10orf96	5'UTR
cg24794228	19	52391166	10	ZNF577;ZNF577;ZNF577;ZNF577;ZNF577	Body;5'UTR;5'UTR;1stExon;1stExon
cg19247475	5	140789745	43	PCDHGA4;PCDHGA9;PCDHGA1;PCDHGB1;PCDHGB6;PCDHGB6;PCDHGB3;PCDHGA6;PCDHGA8;PCDHGA5;PCDHGB4;PCDHGA3;PCDHGA2;PCDHGB2;PCDHGA7;PCDHGB5	Body;Body;Body;Body;1stExon;1stExon;Body;Body;Body;Body;Body;Body;Body;Body;Body;Body
cg00983520	22	51017067	3	CPT1B;CPT1B;CPT1B;C	1stExon;TSS1500;1stExon;

				PT1B;CHKB-CPT1B;CPT1B;CPT1B;CPT1B;CPT1B;CPT1B;CPT1B;CPT1B	5'UTR;Body;1stExon;5'UTR;TSS200;TSS200;1stExon;5'UTR;5'UTR
cg03540175	3	49236946	1	CCDC36;CCDC36;CCDC36	1stExon;5'UTR;5'UTR
cg24332783	11	31818791	3	PAX6;PAX6;PAX6	Body;Body;Body
cg10265016	2	18061319	1	KCNS3	5'UTR
cg18587476	2	24397810	1	C2orf84	TSS200
cg23581541	20	2731176	4	EBF4	Body
cg16987900	5	140207609	12	PCDHA2;PCDHA1;PCDHA1;PCDHA6;PCDHA5;PCDHA6;PCDHA3;PCDHA4;PCDHA6	Body;Body;Body;TSS200;Body;TSS200;Body;Body;TSS200
cg08070771	3	147125758	11	ZIC4;ZIC1	TSS1500;TSS1500
cg16257219	12	3069765	1	TEAD4;TEAD4;TEAD4	5'UTR;5'UTR;5'UTR
cg08380311	19	3435252	1	NFIC;NFIC	Body;Body
cg22328208	8	98289745	1	TSPYL5	1stExon
cg22541830	2	114048868	4	LOC440839	Body
cg19747232	17	32483560	3	ACCN1;ACCN1	1stExon;5'UTR
cg17069396	20	2731102	4	EBF4	Body
cg24369989	15	78933807	1	CHRNB4	TSS1500
cg14227486	4	41867415			
cg13912311	9	127265348	1	NR5A1	Body
cg16872595	18	77710649	1	PQLC1;PQLC1;PQLC1	Body;Body;Body
cg16021896	11	134147525	1	GLB1L3	Body
cg16379671	12	131647826			
cg08387014	11	1848742			
cg12723026	1	110764594	1	KCNC4;KCNC4;KCNC4	Body;Body;Body
cg12744820	6	137814960	2	OLIG3	1stExon
cg27431761	11	8361327			
cg23514016	5	78407564	5	BHMT	TSS200
cg24661016	5	1660205			
cg05708114	9	34624587	1	ARID3C	Body
cg12097989	1	7311176	4	CAMTA1	Body
cg11455444	2	121223947	1	LOC84931	TSS200
cg12635937	4	81184925			
cg15655714	19	51018365	1	ASPDH;ASPDH	TSS1500;TSS1500
cg24968629	22	46770644	1	CELSR1	Body
cg14622069	5	140214328	12	PCDHA6;PCDHA2;PCDHA1;PCDHA7;PCDHA1;PCDHA6;PCDHA5;PCDHA3;PCDHA4;PCDHA7	Body;Body;Body;1stExon;Body;Body;Body;Body;Body;1stExon
cg25779645	20	42965025	1	R3HDML	TSS1500
cg03254465	1	3240227	8	PRDM16;PRDM16	Body;Body
cg11729074	18	22928227	2	ZNF521	Body
cg09538809	5	140782948	43	PCDHGA4;PCDHGA6;PCDHGA1;PCDHGA8;PCDHGA5;PCDHGB1;PCDHGB4;PCDHGA3;PCDHGA2;PCDHGA9;PCDHGA7;PCDHGB2;PCDHGB5;PCDHGA9;PCDHGB3	Body;Body;Body;Body;Body;Body;Body;Body;Body;1stExon;Body;Body;Body;1stExon;Body
cg23009419	3	46618597	1	LRRC2;TDGF1	5'UTR;TSS1500
cg15395354	4	100242862	1	ADH1B	TSS1500
cg07570687	10	102243282	1	WNT8B	3'UTR
cg05016408	5	150326174	1	LOC134466	TSS200
cg15289427	6	24911001	9	FAM65B	5'UTR
cg22913127	19	34624975			
cg15997429	6	168714709	1	DACT2	Body
cg14976741	12	4872921	1	GALNT8	Body
cg14415160	1	47010105			

cg12155969	15	92849000			
cg06687640	5	159560692			
cg00808170	5	140807787	43	PCDHGA4;PCDHGA11;PCDHGA11;PCDHGA9;PCDHGA1;PCDHGB1;PCDHGB8P;PCDHGB6;PCDHGB3;PCDHGB7;PCDHGA6;PCDHGA8;PCDHGA10;PCDHGA5;PCDHGB4;PCDHGA3;PCDHGA2;PCDHGB2;PCDHGA7;PCDHGB5	Body;Body;Body;Body;Body;Body;Body;Body;Body;Body;Body;Body;Body;Body;Body;Body;Body;Body;Body;Body
cg25953930	6	41462541			
cg09695851	17	3907499			
cg17692403	20	42964959	1	R3HDML	TSS1500
cg19409254	13	36431974	4	MIR548F5;DCLK1	Body;Body
cg05329352	10	112838983	1	ADRA2A	1stExon
cg03614193	18	55021542	1	ST8SIA3	Body
cg23295647	14	34269637	1	NPAS3;NPAS3;NPAS3;NPAS3	Body;Body;Body;Body
cg23575754	2	96315200			
cg04986504	11	12031266	1	DKK3;DKK3;DKK3	TSS1500;TSS1500;TSS1500
cg06445016	8	61835848			
cg07158230	7	63391463			
cg00807871	6	37617124	3	MDGA1	Body
cg09547119	19	52391367	10	ZNF577;ZNF577;ZNF577	TSS200;TSS200;TSS200
cg11014124	5	140782297	43	PCDHGA4;PCDHGA6;PCDHGA1;PCDHGA8;PCDHGA5;PCDHGB1;PCDHGB4;PCDHGA3;PCDHGA2;PCDHGA7;PCDHGB2;PCDHGA9;PCDHGB5;PCDHGA9;PCDHGB3	Body;Body;Body;Body;Body;Body;Body;Body;Body;Body;Body;TSS1500;Body;TSS1500;Body
cg02721176	10	118084587	1	C10orf96	Body
cg03925294	4	6247629			
cg06808571	7	150642256	1	KCNH2;KCNH2	3'UTR;3'UTR
cg06777732	12	131118426			
cg26778345	16	8806586	3	ABAT;ABAT	5'UTR;TSS1500
cg00360794	15	41803850	1	LTK;LTK;LTK	Body;Body;Body
cg03596167	16	23724821	1	ERN2	TSS200
cg27571196	1	201253259	1	PKP1;PKP1	Body;Body
cg19070894	5	134823888			
cg18845277	7	48019713	1	HUS1	TSS1500
cg02638589	15	72477069	1	GRAMD2	Body
cg19594666	7	127881280	4	LEP	TSS200

Supplementary Table 3. The 200 most significantly differentially probes between MF mononuclear cells and their healthy age-matched controls. Red indicates hypomethylation and green indicates hypermethylation.

Illumids	Chromosome	Cytosine position at the chromosome	No of probes annotated to the gene	Gene name	Regulatory site annotated
cg05995360	2	236044874			
cg06897661	16	50322074	1	ADCY7;ADCY7	5'UTR;1stExon
cg14926196	6	37616482	5	MDGA1	Body
cg04816311	7	1066650	1	C7orf50;C7orf50;C7orf50	Body;Body;Body
cg17082719	8	144896176	1	SCRIB;SCRIB;MIR937	Body;Body;TSS1500
cg16802439	16	88907184	1	GALNS	Body
cg04340258	19	3398706	1	NFIC;NFIC	Body;Body
cg25921609	17	8379225	1	MYH10	Body
cg17074014	17	3704494	3	ITGAE;ITGAE	1stExon;5'UTR
cg13984928	17	3704574	3	ITGAE	TSS200
cg20765716	17	3704602	3	ITGAE	TSS200
cg23551198	17	3585166	1	P2RX5;P2RX5	Body;Body
cg11883836	3	156838181			
cg01393945	5	10457034	1	ROPN1L	Body
cg14224600	12	117477926	1	TESC;TESC;TESC (=tescalcin)	Body;Body;Body
cg04231085	16	85561302			
cg11204139	17	3907470			
cg01925738	5	140480770	1	PCDHB3	1stExon
cg13642872	15	78527113	1	ACSBG1	TSS1500
cg06468454	17	3591377	1	P2RX5;P2RX5	Body;Body
cg04856396	17	3589140	1	P2RX5;P2RX5	Body;Body
cg21819984	15	101084507	1	LASS3	5'UTR
cg07053114	10	129794994	1	PTPRE	5'UTR
cg02286380	17	4621205	2	ARRB2;ARRB2	Body;Body
cg13466002	17	4621252	2	ARRB2;ARRB2	Body;Body
cg16824643	18	76462312			
cg05890377	2	74357713			
cg02574073	17	46682398	7	LOC404266;LOC404266;HOXB6	Body;Body;TSS200
cg10505257	16	4731639	2	MGRN1;MGRN1;MGRN1;MGRN1	Body;Body;Body;Body
cg01626707	14	105946891			
cg22637435	8	141361108	1	TRAPPC9;TRAPPC9	Body;Body
cg17886399	3	62362660			
cg19787694	19	846117	1	PRTN3 (=ANCA; MBN; MBT; NP4; P29; PR3; ACPA; AGP7; NP-4; PR-3; CANCA; C-ANCA)	Body
cg05825244	20	2730488	1	EBF4	Body
cg02380135	2	149866270	1	KIF5C	Body
cg03062252	6	37616598	5	MDGA1	Body
cg08658787	12	113916646			
cg24833027	8	1897969	1	ARHGEF10	Body
cg15915602	2	114737459			
cg05613718	7	38355100			
cg11460110	6	30530458	1	PRR3;PRR3	3'UTR;3'UTR
cg09667606	6	158507930	1	SYNJ2	Body
cg09246479	22	24891129	1	C22orf45;C22orf45;UPB1	TSS1500;TSS1500;TSS200
cg05715492	7	98991138	1	ARPC1B (=p41-ARC, p40-ARC)	Body

cg27431761	11	8361327			
cg04427254	19	23657425			
cg15084543	1	79472408	3	ELTD1;ELTD1	5'UTR;1stExon
cg00807871	6	37617124	5	MDGA1	Body
cg19112186	22	51016638	6	CPT1B;CPT1B;CPT1B;CHKB-CPT1B;CPT1B;CPT1B;CPT1B;CPT1B	5'UTR;5'UTR;TSS200;Body;5'UTR;5'UTR;5'UTR;5'UTR
cg05941376	5	167836834	1	WWC1;WWC1;WWC1	Body;Body;Body
cg13536060	19	51189671	1	SHANK1	Body
cg24113243	12	132882028	1	GALNT9	Body
cg08616061	5	140753570	2	PCDHGA4;PCDHGA1;PCDHGA6;PCDHGA5;PCDHGB1;PCDHGA3;PCDHGA2;PCDHGA6;PCDHGB2;PCDHGB3	Body;Body;TSS200;Body;Body;Body;Body;TSS200;Body;Body
cg04360793	1	79472361	3	ELTD1;ELTD1	5'UTR;1stExon
cg05156901	22	51016646	6	CPT1B;CPT1B;CPT1B;CHKB-CPT1B;CPT1B;CPT1B;CPT1B;CPT1B	5'UTR;5'UTR;TSS200;Body;5'UTR;5'UTR;5'UTR;5'UTR
cg25645064	3	147096130			
cg01862688	5	140480872	1	PCDHB3	1stExon
cg16776981	17	79428142	1	BAHCC1	Body
cg20045320	11	319555			
cg16791210	2	85581378	1	ELMOD3;ELMOD3;ELMOD3;RETSAT;ELMOD3	TSS1500;TSS1500;TSS1500;Body;TSS1500
cg05016408	5	150326174	2	LOC134466	TSS200
cg06928952	16	87736670	1	LOC100129637	Body
cg24363820	22	51016703	6	CPT1B;CPT1B;CPT1B;CHKB-CPT1B;CPT1B;CPT1B;CPT1B;CPT1B	5'UTR;5'UTR;TSS200;Body;5'UTR;5'UTR;5'UTR;5'UTR
cg19416570	19	58715677	1	ZNF274;ZNF274;ZNF274	Body;Body;5'UTR
cg27260684	16	85063742	1	KIAA0513	5'UTR
cg15996534	5	150325954	2	LOC134466	Body
cg03697308	13	28545566			
cg05701418	6	30131361	4	TRIM15;TRIM15	5'UTR;1stExon
cg14612417	2	95688557			
cg01561304	5	177913485	1	COL23A1	Body
cg10635122	19	52391090	2	ZNF577;ZNF577;ZNF577;ZNF577	Body;5'UTR;5'UTR;1stExon
cg22425467	6	30131189	4	TRIM15;TRIM15	5'UTR;1stExon
cg03548384	16	84403863	1	ATP2C2 (=SPCA2)	Body
cg17179862	17	46681362	7	LOC404266;LOC404266;HOXB6	Body;Body;5'UTR
cg02877240	7	112089798	1	IFRD1;IFRD1	TSS1500;5'UTR
cg14130459	11	60718568	1	SLC15A3;SLC15A3	1stExon;Body
cg06445016	8	61835848			
cg04260676	1	1774322	1	GNB1	5'UTR
cg01042465	7	54765601			
cg17344770	19	3537694	1	C19orf71	TSS1500
cg21488279	5	122434178	1	PRDM6	Body
cg07333191	4	13526769			
cg19711268	14	103367858	1	TRAF3;TRAF3;TRAF3	Body;Body;Body
cg20053110	6	37617864	5	MDGA1	Body
cg15289427	6	24911001	2	FAM65B	5'UTR
cg17280346	3	147126703	2	ZIC1	TSS1500
cg09287328	10	134231487			
cg12570429	17	56345839	1	LPO;LPO;LPO	3'UTR;3'UTR;Body
cg16717713	14	100069657	1	CCDC85C	1stExon
cg04259907	19	43969884	1	LYPD3(=C4.4A)	TSS200

cg09452568	5	54275198	1	ESM1;ESM1	Body;Body
cg06530441	22	51016950	6	CPT1B;CPT1B;CPT1B;CHKB-CPT1B;CPT1B;CPT1B;CPT1B;CPT1B	TSS200;TSS1500;5'UTR;Body;5'UTR;5'UTR;5'UTR;TSS200
cg16270399	18	74257894	1	LOC284276	Body
cg03315662	16	30759178	1	PHKG2	TSS1500
cg00720829	6	30131219	4	TRIM15;TRIM15	5'UTR;1stExon
cg26675289	7	149916700			
cg10906729	17	46682390	7	LOC404266;LOC404266;HOXB6	Body;Body;TSS200
cg21890667	22	30476089	4	HORMAD2	TSS1500
cg06777732	12	131118426			
cg18294691	14	38080669			
cg22879098	5	672845	1	TPPP	Body
cg01443020	17	15653096			
cg18667659	2	99438903	1	C2orf55	Body
cg18471993	6	100917416			
cg05273302	19	2095560	1	C19orf36;MOBKL2A;C19orf36	TSS1500;5'UTR;TSS1500
cg23916496	10	85997314	1	LRIT1	Body
cg18023065	11	94278603	1	FUT4 (=LeX; CD15; ELFT; FCT3A; FUTIV; SSEA-1; FUC-TIV)	1stExon
cg06757399	16	1878010	1	HAGH;FAHD1;FAHD1;FAHD1;HAGH;FAHD1	TSS1500;Body;Body;1stExon;TSS1500;3'UTR
cg05888755	17	46682319	7	LOC404266;LOC404266;HOXB6;HOXB6	Body;Body;1stExon;5'UTR
cg08710629	16	87903292	1	SLC7A5 (=E16; CD98; LAT1; 4F2LC; MPE16; hLAT1; D16S469E)	TSS200
cg12765028	4	13526659			
cg05834845	3	195489306	1	MUC4;MUC4;MUC4	Body;Body;Body
cg22340508	19	22891978			
cg07822928	19	616454	1	HCN2	Body
cg15777760	16	29832180	1	C16orf53;MVP;MVP	3'UTR;5'UTR;5'UTR
cg20087519	10	135379242	1	SYCE1;SYCE1;SYCE1	TSS200;TSS200;5'UTR
cg07336350	16	54322127			
cg20748533	19	51189975	1	SHANK1	Body
cg09419670	9	123605666	1	PSMD5;LOC253039	TSS1500;Body
cg25482900	5	167956911	1	FBLL1	Body
cg27456487	17	56349062	1	MPO	Body
cg27659049	12	115173713			
cg06424065	4	6247640			
cg22541830	2	114048868	1	LOC440839	Body
cg16686158	22	30476098	4	HORMAD2	TSS1500
cg09797202	10	102111107	1	SCD	Body
cg08058191	17	39781130	1	KRT17	TSS1500
cg21714266	6	146350748	1	GRM1;GRM1	Body;Body
cg18891762	5	140746049	1	PCDHGA4;PCDHGA2;PCDHGA5;PCDHGB2;PCDHGA1;PCDHGB1;PCDHGA3;PCDHGA5	Body;Body;1stExon;Body;Body;Body;Body;1stExon
cg02721176	10	118084587	1	C10orf96	Body
cg23749482	17	77901317			
cg00983520	22	51017067	6	CPT1B;CPT1B;CPT1B;CPT1B;CHKB-CPT1B;CPT1B;CPT1B;CPT1B;CPT1B;CPT1B;CPT1B	1stExon;TSS1500;1stExon;5'UTR;Body;1stExon;5'UTR;TSS200;TSS200;1stExon;5'UTR;5'UTR

cg04046669	22	30476206	4	HORMAD2	TSS1500
cg03203197	16	34587120			
cg19828220	17	46682413	7	LOC404266;LOC404266;HOXB6	Body;Body;TSS200
cg00228891	1	207818893	1	CR1L	Body
cg02987928	1	16553456			
cg13327545	10	22623548			
cg07093155	16	9045307	1	USP7	Body
cg07553761	3	160167977	1	TRIM59	TSS1500
cg16257219	12	3069765	1	TEAD4;TEAD4;TEAD4	5'UTR;5'UTR;5'UTR
cg17069396	20	2731102	1	EBF4	Body
cg18384588	22	46463747			
cg17672614	6	166382607	1	C6orf176;C6orf176	Body;Body
cg05162306	14	95826734			
cg14244013	17	59473124			
cg21390082	17	33842255	1	SLFN12L	Body
cg02360514	19	45975990	1	FOSB;FOSB	Body;Body
cg26422458	1	79472452	3	ELTD1;ELTD1	5'UTR;1stExon
cg20100745	8	134307728	1	NDRG1;NDRG1	5'UTR;5'UTR
cg04079760	16	68269694	1	ESRP2	Body
cg03803541	17	46681401	7	LOC404266;LOC404266;HOXB6	Body;Body;5'UTR
cg01024444	19	52222446	1	HAS1	Body
cg14882700	4	4228571	1	OTOP1	1stExon
cg10770023	22	51016644	6	CPT1B;CPT1B;CPT1B;CHKB-CPT1B;CPT1B;CPT1B;CPT1B;CPT1B	5'UTR;5'UTR;TSS200;Body;5'UTR;5'UTR;5'UTR;5'UTR
cg04707519	10	21799314			
cg16848873	17	46682308	7	LOC404266;LOC404266;HOXB6;HOXB6	Body;Body;1stExon;5'UTR
cg12560020	15	93823645			
cg09338032	12	113916609			
cg10399099	16	51169072			
cg10192893	4	41747895	1	PHOX2B	Body
cg26389950	5	140562236	1	PCDHB16	1stExon
cg10783469	19	52391234	2	ZNF577;ZNF577;ZNF577	TSS200;TSS200;TSS200
cg16647921	4	41867533			
cg06110802	4	152020177	1	RPS3A	TSS1500
cg24796644	6	37617956	5	MDGA1	Body
cg02185182	7	2185550	1	MAD1L1;MAD1L1;MAD1L1	Body;Body;Body
cg10378348	1	6244619			
cg08070771	3	147125758	2	ZIC4;ZIC1	TSS1500;TSS1500
cg20959460	5	77253544			
cg08889009	15	59157123			
cg07135405	20	62573077	1	UCKL1;MIR1914	Body;TSS200
cg25351606	6	100917427			
cg17201760	4	206562	2	ZNF876P	Body
cg05592035	17	72462984	1	CD300A	Body
cg15209808	22	30476254	4	HORMAD2	TSS200
cg18740583	19	43202312			
cg02772995	2	161348781	1	RBMS1;RBMS1	Body;Body
cg18306861	14	67879101	1	PLEK2	TSS1500
cg02313331	16	29625259	1	SLC7A5P1	TSS1500
cg01295399	10	50820278	1	SLC18A3;CHAT;CHAT;CHAT	1stExon;TSS1500;5'UTR;TSS1500
cg20116128	6	32161004	1	GPSM3	5'UTR
cg25072733	13	37249481	1	C13orf36	5'UTR
cg01966791	20	62572875	1	MIR1914;UCKL1	Body;Body
cg10375954	1	6419608	1	ACOT7;ACOT7;ACOT7;ACOT7	Body;Body;TSS1500;Body

cg14191466	14	103440432	1	CDC42BPB	Body
cg09979478	8	145925997			
cg11959399	20	741723	1	C20orf54	Body
cg26814075	7	127881298	1	LEP	TSS200
cg27436118	16	4729905	1	MGRN1;MGRN1;MGRN1;MGRN1	Body;Body;Body;Body
cg18274480	5	145713863			
cg06335867	7	8482325	1	NXPH1	Body
cg18283342	5	140235435	2	PCDHA6;PCDHA2;PCDHA1;PCDHA9;PCDHA7;PCDHA1;PCDHA6;PCDHA10;PCDHA5;PCDHA3;PCDHA4;PCDHA10;PCDHA10;PCDHA8	Body;Body;Body;Body;Body;Body;Body;TSS200;Body;Body;Body;TSS200;TSS200;Body
cg03038262	11	315262	1	IFITM1	3'UTR
cg03134157	8	145926089			
cg22068400	2	90016264			
cg19936032	6	24911414	2	FAM65B	TSS1500
cg21584251	5	175488849			
cg11021222	12	97300417	1	NEDD1;NEDD1;NEDD1;NEDD1	TSS1500;TSS1500;TSS1500;TSS1500

Supplementary Table 4. The 200 most significantly differentially probes between MF granulocytes and their healthy age-matched controls. Red indicates hypomethylation and green indicates hypermethylation.

Illumids	Chromosome	Cytosine position at the chromosome	No of probes annotated to the gene	Gene name	Regulatory site annotated
cg19832347	13	24887606	1	C1QTNF9	5'UTR
cg09092525	20	58570133	1	CDH26;CDH26	Body;TSS1500
cg23749482	17	77901317			
cg24263283	15	59587546	2	MYO1E	Body
cg07573872	19	1126342	1	SBNO2;SBNO2	Body;Body
cg02716826	9	33447032	1	SUGT1P1;AQP3	Body;Body
cg09122035	11	319667			
cg14864167	8	66751182	1	PDE7A	Body
cg08101174	10	104196541			
cg12516875	22	46463543			
cg16137564	14	89024217			
cg23570810	11	315102	2	IFITM1	Body
cg22764925	22	24979964	1	GGT1	5'UTR
cg20220678	8	19243209	1	SH2D4A	Body
cg13442016	10	104196339	2	MIR146B	Body
cg16755922	17	80536214	1	FOXK2	Body
cg06635946	22	46470016			
cg06559878	5	156642345	1	ITK	Body
cg18384588	22	46463747			
cg04173586	19	2167496	1	DOT1L	Body
cg06468454	17	3591377	1	P2RX5;P2RX5	Body;Body
cg08818610	6	24910720	3	FAM65B	5'UTR
cg08854834	21	30517941	1	C21orf7	Body
cg24750513	2	127819455	1	BIN1;BIN1;BIN1;BIN1;BIN1;BIN1;BIN1;BIN1;BIN1;BIN1	Body;Body;Body;Body;Body;Body;Body;Body;Body;Body
cg05215830	19	55549826	4	GP6;GP6	TSS200;TSS200
cg19748027	5	154892698			
cg08190125	14	106145435			
cg23796967	19	55549590	4	GP6;GP6	1stExon;1stExon
cg06573254	5	174909270	1	SFXN1	5'UTR
cg12723026	1	110764594	1	KCNC4;KCNC4;KCNC4	Body;Body;Body
cg26549330	22	46471442			
cg03038262	11	315262	2	IFITM1	3'UTR
cg05339605	14	91269861	1	TTC7B	Body
cg08632701	21	37451849			
cg05399785	1	3564031	1	WDR8	Body
cg15857661	10	104196243	2	MIR146B	TSS200
cg20708135	11	76493489	2	TSKU	TSS1500
cg08231710	1	1566687	1	MMP23A;MMP23B	TSS1500;TSS1500
cg23060513	19	13041124	1	FARSA	Body
cg14003931	10	134938469	2	GPR123	Body
cg03697308	13	28545566			
cg20886049	11	76493545	2	TSKU	TSS1500
cg19619585	15	101170019	1	ASB7;ASB7	Body;Body
cg00565412	21	26933554	1	MIR155HG	TSS1500
cg05483571	17	77901224			
cg07298772	2	176306492			
cg12273319	19	50861262			
cg09317239	10	21818060			
cg11202887	13	112076016			
cg17835606	15	48839797	1	FBN1	Body

cg09462576	1	228297873	1	MRPL55;MRPL55;MRPL55;MRPL55;MRPL55;MRPL55;MRPL55;MRPL55	TSS1500;TSS1500;TSS1500;TSS1500;TSS1500;TSS1500;TSS1500;TSS1500
cg17953764	4	48492845	1	ZAR1	1stExon
cg20608990	2	202097607	1	CASP8;CASP8;CASP8;CASP8	TSS1500;TSS1500;TSS1500;TSS1500
cg27558541	1	110350324			
cg22997040	8	123875223	1	ZHX2	5'UTR
cg12873919	20	30309627	3	BCL2L1;BCL2L1	Body;Body
cg23005227	3	50645426	2	CISH;CISH	Body;Body
cg25645064	3	147096130			
cg13081213	7	26714757	1	SKAP2	Body
cg16116203	12	6876922	1	PTMS	Body
cg19416570	19	58715677	1	ZNF274;ZNF274;ZNF274	Body;Body;5'UTR
cg15701612	11	130253770			
cg08387014	11	1848742			
cg12218747	21	37451666			
cg03089870	6	17988960	1	KIF13A;KIF13A;KIF13A;KIF13A	TSS1500;TSS1500;TSS1500;TSS1500
cg19653417	12	132654924			
cg00946921	14	106025021			
cg13297560	15	99320054	1	IGF1R	Body
cg06633081	14	55120781	1	SAMD4A;SAMD4A	Body;Body
cg12673429	18	11150043	1	FAM38B	TSS1500
cg14778437	13	100148404			
cg03254465	1	3240227	1	PRDM16;PRDM16	Body;Body
cg04427254	19	23657425			
cg21609584	2	101508685	1	NPAS2	5'UTR
cg21781157	20	47874111	2	ZNFX1	Body
cg13989999	20	30309717	3	BCL2L1;BCL2L1	Body;Body
cg01242348	14	23586886	2	CEBPE (=CRP1; C/EBP-epsilon)	Body
cg18115721	11	73567838	1	MRPL48	Body
cg08220120	5	138727711	1	LOC389333;LOC389333	1stExon;3'UTR
cg16359657	1	41119931	1	RIMS3	5'UTR
cg15252509	18	56337165	1	MALT1;MALT1	TSS1500;TSS1500
cg21856784	6	30131214	5	TRIM15;TRIM15	5'UTR;1stExon
cg23916496	10	85997314	1	LRIT1	Body
cg14289429	9	134139878	1	FAM78A	Body
cg01332882	20	47874155	2	ZNFX1	Body
cg14116129	5	1140748			
cg02003183	14	103415882	1	CDC42BPB	Body
cg17280346	3	147126703	1	ZIC1	TSS1500
cg11070172	14	23586812	2	CEBPE	Body
cg13006681	5	158476743	1	EBF1	Body
cg19821713	15	96909816			
cg15724534	2	211018363	1	C2orf67	Body
cg15618978	3	160167990	2	TRIM59	TSS1500
cg18518074	11	64642316	1	EHD1	Body
cg17939935	15	59646854	2	MYO1E	Body
cg00399027	16	85676861	1	KIAA0182;KIAA0182	5'UTR;Body
cg11562153	6	28493500	1	GPX5;GPX5	TSS1500;TSS1500
cg17508591	5	140214138	1	PCDHA6;PCDHA2;PCDHA1;PCDHA7;PCDHA1;PCDHA6;PCDHA5;PCDHA3;PCDHA4;PCDHA7	Body;Body;Body;1stExon;Body;Body;Body;Body;Body;1stExon
cg14903689	21	46875218	2	COL18A1;COL18A	TSS1500;Body;TSS150

				1;COL18A1	0
cg17485681	10	73565625	1	CDH23	Body
cg09338032	12	113916609			
cg08658787	12	113916646			
cg03672272	22	46470191			
cg08317738	16	89034292	2	CBFA2T3	Body
cg11883836	3	156838181			
cg00548098	16	11295667			
cg10647833	6	167047842	1	RPS6KA2	Body
cg05491854	6	24910562	3	FAM65B	5'UTR
cg03497652	16	4751569	1	ANKS3	Body
cg07463541	5	140181074	1	PCDHA2;PCDHA1;PCDHA3;PCDHA3;PCDHA1	Body;Body;1stExon;1stExon;Body
cg11339839	17	77901129			
cg18687085	18	56067462	1	NEDD4L;NEDD4L;NEDD4L;NEDD4L;NEDD4L;NEDD4L;NEDD4L;NEDD4L;NEDD4L	3'UTR;3'UTR;3'UTR;3'UTR;3'UTR;3'UTR;3'UTR;3'UTR;3'UTR
cg06757928	1	2811411			
cg04360793	1	79472361	3	ELTD1;ELTD1	5'UTR;1stExon
cg02772995	2	161348781	2	RBMS1;RBMS1	Body;Body
cg27571196	1	201253259		PKP1;PKP1	Body;Body
cg00314029	2	161348665	2	RBMS1;RBMS1	Body;Body
cg23715749	1	37413867	1	GRIK3	Body
cg17801352	2	1749076	1	PXDN	TSS1500
cg18132076	6	1970710	1	GMDS	Body
cg19092396	1	1143751			
cg16717713	14	100069657	2	CCDC85C	1stExon
cg20116128	6	32161004	1	GPSM3	5'UTR
cg20663365	2	127729097			
cg05765605	16	69360307			
cg11254700	19	53561386			
cg15589415	7	157261698			
cg07586026	1	12600225			
cg00720829	6	30131219	5	TRIM15;TRIM15	5'UTR;1stExon
cg26262840	14	90767686	1	C14orf102;C14orf102	Body;Body
cg07822928	19	616454	1	HCN2	Body
cg11204139	17	3907470			
cg05016408	5	150326174	1	LOC134466	TSS200
cg02150077	16	87098542			
cg10558233	8	94892613			
cg12422154	6	30130819	5	TRIM15	TSS200
cg05406635	14	100069666	2	CCDC85C	1stExon
cg21886042	13	112076036			
cg09676013	6	33080500	1	HLA-DPB2	Body
cg24968629	22	46770644	1	CELSR1	Body
cg12804791	11	126286828			
cg16731240	19	52391250	9	ZNF577;ZNF577;ZNF577	TSS200;TSS200;TSS200
cg15782228	20	60932415	1	LAMA5	Body
cg21585138	3	50645106	2	CISH;CISH	Body;Body
cg05306310	21	43186286	1	RIPK4	Body
cg06633438	19	6272158	1	MLLT1	Body
cg18166636	5	178956931			
cg26422458	1	79472452	3	ELTD1;ELTD1	5'UTR;1stExon
cg16265717	1	65993149		LEPR;LEPR;LEPR	5'UTR;5'UTR;5'UTR
cg17572056	3	195947062	1	OSTalpha	Body
cg17714703	19	4912221	1	UHRF1;UHRF1	Body;Body
cg00084577	16	30721139	1	SNORA30;SRCAP	TSS1500;Body

cg22226592	1	109439213	1	GPSM2	Body
cg04433322	17	42346894	1	SLC4A1	TSS1500
cg18181703	17	76354621	1	SOCS3	Body
cg07938847	4	82126349	1	PRKG2	TSS200
cg15289427	6	24911001	3	FAM65B	5'UTR
cg25953130	10	63753550	1	ARID5B	Body
cg10783469	19	52391234	9	ZNF577;ZNF577;ZNF577	TSS200;TSS200;TSS200
cg07553761	3	160167977	2	TRIM59	TSS1500
cg24794228	19	52391166	9	ZNF577;ZNF577;ZNF577;ZNF577;ZNF577	Body;5'UTR;5'UTR;1stExon;1stExon
cg05089897	21	38886664	1	DYRK1A;DYRK1A;DYRK1A;DYRK1A;DYRK1A	3'UTR;3'UTR;3'UTR;3'UTR;3'UTR
cg01205267	13	47126300	2	LRCH1;LRCH1;LRCH1	TSS1500;TSS1500;TSS1500
cg04220636	16	89034088	2	CBFA2T3	Body
cg17962090	2	127728910			
cg12116137	17	1576449	1	PRPF8	Body
cg18812353	15	56385430	1	RFX7	3'UTR
cg06530441	22	51016950	1	CPT1B;CPT1B;CPT1B;CHKB-CPT1B;CPT1B;CPT1B;CPT1B;CPT1B	TSS200;TSS1500;5'UTR;Body;5'UTR;5'UTR;5'UTR;TSS200
cg10172979	14	88621579			
cg13486805	19	39738664			
cg03313140	2	61403611	1	AHSA2	TSS1500
cg16288101	14	88621538			
cg22446998	5	155701819			
cg02386311	16	12073297	1	RUNDC2A	Body
cg13642872	15	78527113	1	ACSBG1	TSS1500
cg18845417	2	95745976			
cg04389994	10	74590936	1	CCDC109A	Body
cg02895948	1	208204062	1	PLXNA2	Body
cg10192893	4	41747895	1	PHOX2B	Body
cg04864807	2	121412139			
cg20045320	11	319555			
cg01713272	18	12911711			
cg01024444	19	52222446	1	HAS1	Body
cg16379910	14	78636730			
cg20987067	8	8747297	1	MFHAS1	Body
cg08616061	5	140753570	1	PCDHGA4;PCDHGA1;PCDHGA6;PCDHGA5;PCDHGB1;PCDHGA3;PCDHGA2;PCDHGA6;PCDHGB2;PCDHGB3	Body;Body;TSS200;Body;Body;Body;Body;TSS200;Body;Body
cg11186858	17	75096382			
cg08257009	14	94812770			
cg16121744	21	46875149	2	COL18A1;COL18A1;COL18A1	TSS1500;Body;TSS1500
cg14882700	4	4228571	1	OTOP1	1stExon
cg04387347	16	88537187	2	ZFPM1	Body
cg13546414	14	37053664			
cg03423077	1	87599262	1	LOC339524;LOC339524;LOC339524;LOC339524;LOC339524	Body;Body;Body;Body;Body
cg20192747	18	44774846			
cg11379081	1	209405050			
cg26985878	17	79799549			

cg21488279	5	122434178	1	PRDM6	Body
cg07336350	16	54322127			
cg14244013	17	59473124			

Supplementary Table 5. The 308 differentially methylated probes associated with the *ASXL1* mutated DNA methylation signature probes in the CD34+ cell population. Red indicates hypomethylation and green indicates hypermethylation.

Illumids	Chr	Cytosine position at the chromosome	No of probes annotated to the gene	Gene name	Regulatory site annotated	H3K4me1*	H3K4me3*	H3K27me3*
cg01337207	6	32063835	25	TNXB	Body	0	0	1
cg01485117	6	32064677	25	TNXB	Body	0	0	1
cg02989255	6	32063774	25	TNXB	Body	0	0	1
cg03130418	6	32063439	25	TNXB	Body	0	0	1
cg03556669	6	32064497	25	TNXB	Body	0	0	1
cg04753078	6	32063619	25	TNXB	Body	0	0	1
cg07481886	6	32014510	25	TNXB	Body;TSS1500	0	0	0
cg07524919	6	32063901	25	TNXB	Body	0	0	1
cg08650890	6	32064613	25	TNXB	Body	0	0	1
cg10365886	6	32063874	25	TNXB	Body	0	0	1
cg12694372	6	32064582	25	TNXB	Body	0	0	1
cg13400512	6	32064578	25	TNXB	Body	0	0	1
cg13698691	6	32064660	25	TNXB	Body	0	0	1
cg14630748	6	32014484	25	TNXB	Body;TSS1500	0	0	0
cg14737484	6	32054561	25	TNXB	Body	0	0	0
cg15014577	6	32064785	25	TNXB	Body	0	0	1
cg15196197	6	32064573	25	TNXB	Body	0	0	1
cg15793329	6	32064749	25	TNXB	Body	0	0	1
cg18460422	6	32063553	25	TNXB	Body	0	0	1
cg21289669	6	32064764	25	TNXB	Body	0	0	1
cg21642103	6	32064656	25	TNXB	Body	0	0	1
cg24882324	6	32064508	25	TNXB	Body	0	0	1
cg26266427	6	32063838	25	TNXB	Body	0	0	1
cg26695758	6	32063607	25	TNXB	Body	0	0	1
cg27387193	6	32064032	25	TNXB	Body	0	0	1
cg14476700	4	46126261	3	GABRG1	TSS200	0	0	1
cg16091553	4	46126245	3	GABRG1	TSS200	0	0	1
cg16332065	4	46126253	3	GABRG1	TSS200	0	0	1
cg03743584	10	135160908	3	PRAP1	1stExon;1stExon;5'UTR;5'UTR	0	0	1
cg10742801	10	135161047	3	PRAP1	Body;Body	0	0	1
cg15549126	10	135160910	3	PRAP1	1stExon;1stExon;5'UTR;5'UTR	0	0	1
cg02482603	1	174843754	3	RABGAP1L	TSS1500;Body	1	1	0
cg04858586	1	174843971	3	RABGAP1L	TSS1500;Body	1	1	0
cg05702218	1	174843909	3	RABGAP1L	TSS1500;Body	1	1	0
cg00774088	15	26109245	2	ATP10A	TSS1500	0	1	1
cg07470694	15	26109249	2	ATP10A	TSS1500	0	1	1
cg02501779	20	54579355	2	CBLN4	5'UTR;1stExon	0	0	1
cg20779964	20	54580070	2	CBLN4	TSS200	0	0	1
cg01310330	7	1787194	2	ELFN1	3'UTR	0	0	0
cg15050051	7	1782000	2	ELFN1	5'UTR	1	1	0
cg01815645	6	32548627	2	HLA-DRB1	Body	0	0	0
cg20022036	6	32549496	2	HLA-DRB1	Body	0	0	0
cg00595030	19	10398582	2	ICAM4	3'UTR;Body;Body	1	1	1
cg19298774	19	10398798	2	ICAM4	3'UTR;3'UTR;Body	1	1	1
cg04206351	11	49582375	2	LOC440040	Body	0	0	1
cg05259545	11	49583127	2	LOC440040	Body	0	0	1
cg01688688	5	140614000	2	PCDHB18	Body	0	1	1
cg25603636	5	140613998	2	PCDHB18	Body	0	1	1
cg08926642	1	7887455	2	PER3	Body	0	1	0
cg17724687	1	7887346	2	PER3	Body	0	1	0
cg05546296	3	50376006	2	RASSF1	TSS1500;TSS1500;B	1	1	0

					ody;Body			
cg20119308	3	50376000	2	RASSF1	TSS1500;TSS1500;Body;Body	1	1	0
cg02655630	20	44658941	2	SLC12A5	Body;Body	0	0	1
cg22752533	20	44657948	2	SLC12A5	1stExon;5'UTR;Body	0	0	1
cg03815358	6	84419352	2	SNAP91	TSS1500;TSS1500	1	1	1
cg05384102	6	84419329	2	SNAP91	TSS1500;TSS1500	1	1	1
cg03234813	21	45811363	2	TRPM2	Body	0	0	0
cg27477277	21	45811432	2	TRPM2	Body	0	0	0
cg08979515	3	111697575	1	ABHD10	TSS1500	1	1	0
cg26334023	17	47287492	1	ABI3	TSS200;TSS1500;TSS200	1	1	0
cg02925039	12	43945213	1	ADAMTS20	Body	0	0	1
cg05160197	1	203135890	1	ADORA1	3'UTR;3'UTR	0	0	0
cg23763137	22	24823509	1	ADORA2A	TSS200	1	0	0
cg03315407	5	14810180	1	ANKH	Body	1	1	0
cg03235179	5	10566165	1	ANKRD33B	Body	1	1	0
cg14780070	1	17951330	1	ARHGEF10L	Body;Body	0	0	0
cg25953130	10	63753550	1	ARID5B	Body	1	0	0
cg19698273	19	3908397	1	ATCAY	Body	0	0	0
cg26788180	6	32097420	1	ATF6B	TSS1500;TSS1500;Body	0	0	0
cg25292663	17	79386969	1	BAHCC1	Body	1	1	0
cg16126665	14	99711752	1	BCL11B	Body;Body	0	0	1
cg17394304	3	187453973	1	BCL6	5'UTR;5'UTR;1stExon;TSS1500	1	0	1
cg15486454	3	133191270	1	BFSP2	Body	0	0	0
cg12571928	2	242499273	1	BOK	Body	0	1	1
cg09997546	11	8931473	1	C11orf17	TSS1500;TSS1500;5'UTR	0	1	1
cg05763097	14	103569340	1	C14orf73	Body	1	0	1
cg27000944	17	74721842	1	C17orf95	TSS1500;Body;Body	1	1	0
cg22402467	12	7168056	1	C1S	1stExon;1stExon;5'UTR;5'UTR	0	0	0
cg00397859	20	13768987	1	C20orf7	Body;Body;Body	0	0	0
cg16746362	6	31692375	1	C6orf25	Body;Body;Body;Body;Body;Body	0	0	0
cg03382797	9	135755363	1	C9orf98	TSS1500;5'UTR	1	1	0
cg24637035	12	120445227	1	CCDC64	Body	0	0	0
cg09554443	1	167487762	1	CD247	1stExon;5'UTR;1stExon;5'UTR	0	0	0
cg13530039	11	62689557	1	CHRM1	TSS1500	0	0	1
cg26446133	18	72167187	1	CNDP2	5'UTR;1stExon;5'UTR	1	1	0
cg13647382	6	75912547	1	COL12A1	5'UTR;5'UTR	1	0	1
cg08572214	4	73936052	1	COX18	TSS1500	1	1	0
cg11694519	11	111783563	1	CRYAB	TSS1500;1stExon	0	0	1
cg08980382	16	31580153	1	CSDAP1	Body	0	1	0
cg00520378	16	88773576	1	CTU2	Body;Body;TSS1500	1	1	0
cg00160914	1	112299263	1	DDX20	Body;TSS1500	1	1	0
cg02814805	3	122514629	1	DIRC2	Body	1	1	0
cg13114315	21	37546467	1	DOPEY2	Body	1	0	0
cg20739526	7	154585989	1	DPP6	Body;Body;Body	0	0	1
cg04311403	1	236559646	1	EDARADD	Body;Body	1	1	0
cg10014308	2	73518221	1	EGR4	3'UTR	1	0	1
cg00926318	20	25177340	1	ENTPD6	5'UTR;Body	1	1	0
cg06433816	9	140311437	1	EXD3	5'UTR	0	0	0
cg04370247	10	126308552	1	FAM53B	3'UTR	1	1	0
cg13843613	1	177140126	1	FAM5B	TSS1500	0	1	1
cg19924352	8	124195402	1	FAM83A	1stExon;1stExon	0	0	0
cg21526019	19	17877577	1	FCHO1	Body;Body;Body;Body	1	0	0

cg26758396	20	6104274	1	FERMT1	TSS200	1	1	0
cg02565993	11	27015592	1	FIBIN	TSS200	0	0	1
cg02987906	4	54243303	1	FIP1L1	TSS1500;TSS1500;TSS1500	1	1	0
cg13167816	14	24601808	1	FITM1	Body	1	0	0
cg03680338	6	22043967	1	FLJ22536	Body	0	0	0
cg27417316	9	2623684	1	FLJ35024	TSS1500;Body;Body	1	1	1
cg11648730	5	92907151	1	FLJ42709	TSS1500;Body;Body	0	0	1
cg13944838	5	179740914	1	GFPT2	Body	0	1	1
cg19666600	12	110907609	1	GPN3	TSS1500;Body	1	1	0
cg03206445	9	140062197	1	GRIN1	3'UTR;3'UTR;Body	1	0	1
cg08616516	11	88796092	1	GRM5	5'UTR	0	0	1
cg07792871	6	29942706	1	HCG9	TSS200	0	0	0
cg07616394	10	94452554	1	HHEX	Body	1	1	0
cg15157455	1	149400292	1	HIST2H2BF	TSS1500	0	1	1
cg21758773	6	30458730	1	HLA-E	Body	0	0	0
cg03757784	16	22826113	1	HS3ST2	1stExon	0	0	1
cg23622369	17	40706682	1	HSD17B1	Body	0	1	0
cg14240353	12	102873312	1	IGF1	Body;Body;Body;TSS1500	1	1	0
cg05323345	11	2163174	1	IGF2AS	Body;Body;5'UTR;TSS1500;Body	0	0	1
cg10978799	1	206945924	1	IL10	TSS200	1	0	0
cg01294808	5	3599686	1	IRX1	Body	0	0	1
cg24725263	12	56101328	1	ITGA7	1stExon;Body;1stExon	1	1	1
cg08129084	15	40713422	1	IVD	3'UTR;3'UTR	0	0	0
cg19658332	22	39097857	1	JOSD1	TSS1500	1	1	0
cg10148473	12	75601824	1	KCNC2	5'UTR;5'UTR;5'UTR	0	0	1
cg01385052	8	140643119	1	KCNK9	Body	1	1	1
cg05373457	8	99439948	1	KCNS2	5'UTR	1	0	1
cg18163342	14	105332627	1	KIAA0284	5'UTR;5'UTR	0	0	1
cg09497409	19	8275083	1	LASS4	5'UTR	1	1	0
cg24332770	1	152658287	1	LCE2B	TSS1500	0	0	0
cg24631526	20	62367961	1	LIME1	TSS200	1	1	0
cg14501219	17	62775713	1	LOC146880	Body;Body	1	0	1
cg15887459	11	18230903	1	LOC494141	Body;Body;Body	0	0	1
cg26112661	1	53704558	1	MAGOH	TSS1500	1	1	0
cg24591861	15	66697173	1	MAP2K1	Body	1	1	0
cg06946838	7	114563609	1	MDFIC	3'UTR;Body;Body	0	1	0
cg20687414	3	44155764	1	MIR138-1	Body	0	0	1
cg17796010	20	26188963	1	MIR663	TSS200	0	1	1
cg25588852	2	216877276	1	MREG	Body	0	1	0
cg21691367	6	151325642	1	MTHFD1L	Body	1	1	0
cg17314538	4	187476837	1	MTNR1A	TSS1500	0	0	1
cg00425764	16	15949616	1	MYH11	5'UTR;5'UTR;5'UTR;5'UTR	0	0	1
cg12178669	10	51566202	1	NCOA4	5'UTR;5'UTR;5'UTR	1	1	0
cg22047387	12	55413872	1	NEUROD4	1stExon;5'UTR	0	0	1
cg07042144	19	54327404	1	NLRP12	1stExon	1	1	0
cg20327163	15	96877194	1	NR2F2	Body;Body;5'UTR;5'UTR	0	0	1
cg04899492	6	24126312	1	NRSN1	TSS200	0	0	1
cg07124642	17	39991813	1	NT5C3L	Body	1	1	0
cg25293325	17	873413	1	NXN	Body	0	0	1
cg14271729	14	57858383	1	NAA30	Body	1	1	0
cg03340878	6	27925166	1	OR2B6	1stExon	1	0	1
cg07962315	9	77704473	1	OSTF1	Body;TSS1500;TSS1500;TSS1500	1	1	0
cg21501724	20	9818832	1	PAK7	5'UTR;5'UTR	0	0	1
cg12011136	7	4901750	1	PAPOLB	TSS200;Body	0	1	1
cg17117459	13	25085405		PARP4	5'UTR	1	1	0

cg02004851	5	140207460	1	PCDHA2	Body;Body;Body;TSS200;Body;TSS200;Body;Body;TSS200	0	0	1
cg19990022	5	140430619	1	PCDHB1	TSS1500	0	0	1
cg03349953	5	140515207	1	PCDHB5	1stExon	0	1	1
cg13912090	5	95767863	1	PCSK1	Body	0	0	1
cg27355501	6	33256078	1	PFDN6	TSS1500;Body;Body	0	0	0
cg04153991	3	111577705	1	PHLDB2	TSS1500;Body;TSS1500;TSS1500	0	1	1
cg23548151	12	130823838	1	PIWIL1	5'UTR	0	0	0
cg02392228	19	4523345	1	PLIN5	3'UTR	0	0	0
cg01189606	3	48471901	1	PLXNB1	TSS1500;TSS1500	1	1	0
cg04284140	7	44163975	1	POLD2	TSS1500;TSS1500	1	1	0
cg00997604	8	43146894	1	POTEA	TSS1500;TSS1500	0	0	1
cg20073050	4	106395643	1	PPA2	TSS1500;TSS1500;TSS1500;TSS1500;TSS1500	1	1	0
cg19526455	17	37787862	1	PPP1R1B	Body;Body	1	0	0
cg26529371	19	12911983	1	PRDX2	Body;Body	1	1	0
cg06532880	5	176731545	1	PRELID1	Body;TSS1500;TSS1500	1	1	0
cg16190265	7	650249	1	PRKAR1B	Body;Body;Body;Body;Body;Body	1	0	0
cg05696092	19	50084283	1	PRRG2	TSS1500;TSS1500	1	1	0
cg09309269	17	30770961	1	PSMD11	TSS1500	1	1	1
cg04234016	12	7062109	1	PTPN6	Body;Body;Body	1	1	0
cg11958644	5	130872422	1	RAPGEF6	Body;Body;Body;Body;Body;Body	0	1	0
cg23956565	5	73066639	1	RGNEF	Body	1	0	0
cg00876678	16	2319586	1	RNPS1	TSS1500	1	0	0
cg16767880	17	113262	1	RPH3AL	Body	0	0	0
cg13217373	6	167275395	1	RPS6KA2	Body	1	0	1
cg23289581	17	1840546	1	RTN4RL1	Body	0	0	0
cg01733438	17	75276069	1	SEPT9	TSS1500	0	0	0
cg23542968	14	36983129	1	SFTA3	TSS200	0	0	1
cg02904344	6	134498979	1	SGK1	Body;1stExon;5'UTR	1	1	1
cg01081083	22	25202766	1	SGSM1	Body;Body;Body;Body	1	1	0
cg16963852	3	150479084	1	SIAH2	Body	1	1	0
cg17761772	19	51627208	1	SIGLEC9	TSS1500	0	0	0
cg24000908	5	1113358	1	SLC12A7	TSS1500	0	0	0
cg17132030	19	17599784	1	SLC27A1	Body	1	1	0
cg13910439	9	2022036	1	SMARCA2	5'UTR;5'UTR	1	1	0
cg09204187	2	130940668	1	SMPD4	TSS1500;TSS1500;TSS1500;TSS1500;TSS1500;TSS1500;TSS1500;Body	1	1	0
cg22356061	1	227954102	1	SNAP47	Body	1	0	0
cg22327175	11	121323934	1	SORL1	Body	1	1	0
cg12623536	10	73848817	1	SPOCK2	TSS200;TSS200	1	1	1
cg11210813	20	35972385	1	SRC	TSS1500	0	0	0
cg15484375	11	18287647	1	SAA1	TSS200;TSS200	0	0	0
cg16288089	7	97361408	1	TAC1	5'UTR;1stExon;1stExon;1stExon;5'UTR;5'UTR;1stExon;5'UTR	1	0	1
cg14707053	6	42019127	1	TAF8	Body	1	1	0
cg00326464	6	10404084	1	*TFAP2A*	Body;Body;Body	1	1	1
cg24845329	19	376532	1	THEG	TSS1500;TSS1500	0	0	1
cg12029105	12	120032623	1	TMEM233	Body	0	0	1
cg12054453	17	57915717	1	TMEM49	Body	0	1	0
cg23629150	8	144416404	1	TOP1MT	Body	1	1	0
cg14601038	2	1481097	1	TPO	Body;Body;Body;Bo	0	0	1

					dy			
cg05936516	5	114507066	1	TRIM36	Body;Body	1	1	1
cg23427912	4	189059510	1	TRIML1	TSS1500	0	0	0
cg00699986	20	25057444	1	VSX1	Body	0	0	1
cg13699009	12	122356056	1	WDR66	TSS1500	1	1	0
cg17885791	6	149771845	1	ZC3H12D	Body	1	1	1
cg09241332	3	147126638	1	ZIC1	TSS1500	0	0	1
cg07948034	13	100635905	1	ZIC2	Body	0	0	1
cg22202121	6	87866253	1	ZNF292	Body	1	1	0
cg27213549	10	44102591	1	ZNF485	Body	1	1	0
cg11824827	16	31075547	1	ZNF668	Body	1	1	0
cg18693673	19	58629975	1	ZSCAN18	TSS200;TSS200	0	0	1
cg00036788	4	54974682				1	0	1
cg00815399	7	158750607				1	1	1
cg01202519	11	85645743				0	0	1
cg02019125	3	156323952				1	1	0
cg02093732	15	30517601				0	0	1
cg02778237	6	72298242				0	0	1
cg03052128	12	85671811				0	0	1
cg03168896	3	44036098				1	0	1
cg03255953	3	127056837				0	0	1
cg03296200	2	629022				0	0	1
cg03582285	17	40700314				0	1	0
cg03692563	11	31846414				0	0	1
cg03830181	13	20676097				0	0	1
cg04206699	8	70855057				1	1	1
cg05590265	6	133889379				0	0	1
cg05684715	12	130641648				0	0	1
cg05905179	15	73313741				0	0	0
cg06197836	1	39283522				1	0	1
cg06224587	4	6540449				0	0	1
cg06246357	6	1524494				1	1	1
cg06573787	8	143070187				0	0	1
cg06710769	20	58509559				1	1	0
cg06982272	20	2188575				1	1	1
cg07149609	1	170630734				0	0	1
cg07173635	9	971480				0	0	1
cg07671678	8	70855046				1	1	1
cg07694864	17	40700509				0	1	0
cg07856962	21	44864361				0	0	1
cg08214455	16	33817457				0	0	1
cg08566455	2	130971164				0	0	1
cg08927145	16	86321899				0	0	1
cg09208331	21	44864004				0	0	1
cg09985260	15	24346942				0	0	1
cg10014005	17	22051978				0	0	0
cg10079374	7	158750417				1	0	1
cg10193711	14	22917797				1	0	0
cg10779492	1	106623781				0	0	1
cg11491998	6	30419493				0	0	0
cg11710851	12	130765858				0	0	1
cg11945929	7	158750384				1	0	1
cg12580930	11	2013668				0	0	0
cg12823839	7	155175340				1	0	0
cg13283952	2	121412005				0	0	1
cg13391313	17	80330415				1	0	1
cg13420413	1	2347015				1	0	0
cg13692446	13	112759719				0	0	0
cg13790618	19	30715001				0	0	1
cg14283758	4	299370				1	0	1
cg14317384	8	216788				0	0	0

cg14398295	15	30337018				0	0	1
cg14459158	9	96720562				0	0	1
cg14685796	5	50265660				1	1	1
cg14753872	6	33873641				1	1	0
cg15299057	8	70855146				1	1	1
cg16021678	13	55475951				0	0	0
cg16236785	12	120809271				0	0	1
cg16291880	6	50674399				0	0	1
cg16458596	14	99584812				0	0	1
cg17397150	6	101840567				0	0	1
cg17680767	5	73623208				1	0	1
cg17842822	6	1310485				0	0	1
cg17932096	4	58060773				0	0	1
cg18290233	5	92931173				0	0	1
cg18973863	13	97794113				0	0	1
cg19526659	19	57154862				0	0	1
cg19731055	19	5892313				1	0	1
cg20026939	18	44788898				0	0	1
cg20300343	1	149719461				0	0	1
cg20461188	10	102322354				0	0	0
cg20657674	5	180600912				0	0	1
cg20859731	20	36226820				0	0	1
cg21074656	11	134284116				0	0	1
cg21739816	4	3746443				0	0	1
cg21854286	17	40700858				1	1	0
cg22356722	2	176990921				0	0	0
cg22481770	3	156324118				1	1	0
cg22864077	2	131185333				0	1	1
cg22913127	19	34624975				1	1	0
cg23351289	19	34849945				0	0	0
cg23883058	2	5813866				0	0	1
cg24114014	6	28584167				0	0	1
cg24495585	4	56659985				1	1	1
cg24907011	8	24855940				0	0	1
cg25243082	4	40267141				1	0	0
cg26098768	20	2187533				1	0	1
cg26405475	3	156324038				1	1	0
cg26408364	12	53108600				0	0	1
cg26537209	16	54324187				0	0	1
cg26613361	16	31830668				0	0	1
cg26860935	4	1407989				0	0	0
cg27088072	2	242481453				0	0	1
cg27432085	1	11958572				0	0	1

* Chip-seq peaks p<0.01; 0 indicates no enrichment, 1 indicates enrichment

Supplementary Table 6. The 281 differentially methylated probes associated with the *ASXL1* mutated DNA methylation signature probes in the granulocytes population. Red indicates hypomethylation and green indicates hypermethylation.

Illumids	Chromosome	Cytosine position at the chromossome	No of probes annotated to the gene	Gene Name	Regulatory site annotated
cg21337909	6	32063459	37	TNXB	Body
cg01992382	6	32064212	37	TNXB	Body
cg20414186	6	32064491	37	TNXB	Body
cg02989255	6	32063774	37	TNXB	Body
cg10890302	6	32064246	37	TNXB	Body
cg24882324	6	32064508	37	TNXB	Body
cg10923662	6	32064258	37	TNXB	Body
cg21342636	6	32064671	37	TNXB	Body
cg03556669	6	32064497	37	TNXB	Body
cg04753078	6	32063619	37	TNXB	Body
cg07524919	6	32063901	37	TNXB	Body
cg26695758	6	32063607	37	TNXB	Body
cg27081346	6	32064692	37	TNXB	Body
cg16834823	6	32064218	37	TNXB	Body
cg21642103	6	32064656	37	TNXB	Body
cg18330047	6	32064783	37	TNXB	Body
cg01485117	6	32064677	37	TNXB	Body
cg00525277	6	32064239	37	TNXB	Body
cg15196197	6	32064573	37	TNXB	Body
cg18460422	6	32063553	37	TNXB	Body
cg15014577	6	32064785	37	TNXB	Body
cg10365886	6	32063874	37	TNXB	Body
cg26266427	6	32063838	37	TNXB	Body
cg13698691	6	32064660	37	TNXB	Body
cg21289669	6	32064764	37	TNXB	Body
cg00872984	6	32063991	37	TNXB	Body
cg13400512	6	32064578	37	TNXB	Body
cg15265085	6	32064588	37	TNXB	Body
cg08650890	6	32064613	37	TNXB	Body
cg12694372	6	32064582	37	TNXB	Body
cg03130418	6	32063439	37	TNXB	Body
cg01337207	6	32063835	37	TNXB	Body
cg25375757	6	32014739	37	TNXB	Body;TSS1500
cg09423875	6	32014663	37	TNXB	Body;TSS1500
cg14630748	6	32014484	37	TNXB	Body;TSS1500

cg08972588	6	32014674	37	TNXB	Body;TSS1500
cg13409674	6	32014476	37	TNXB	Body;TSS1500
cg04725166	1	7887271	5	PER3	Body
cg17328665	1	7887199	5	PER3	Body
cg08926642	1	7887455	5	PER3	Body
cg12258811	1	7844735	5	PER3	TSS200
cg09168692	1	7887560	5	PER3	Body
cg06132876	6	31692080	3	C6orf25	Body;Body;Body;Body
cg26114961	6	31692295	3	C6orf25	Body;Body;Body;Body
cg16746362	6	31692375	3	C6orf25	Body;Body;Body;Body
cg04858586	1	174843971	3	RABGAP1L	TSS1500;Body
cg05702218	1	174843909	3	RABGAP1L	TSS1500;Body
cg02482603	1	174843754	3	RABGAP1L	TSS1500;Body
cg05754719	21	45811549	3	TRPM2	Body
cg27477277	21	45811432	3	TRPM2	Body
cg03234813	21	45811363	3	TRPM2	Body
cg02925039	12	43945213	2	ADAMTS20	Body
cg11751806	12	43945680	2	ADAMTS20	1stExon
cg14476700	4	46126261	2	GABRG1	TSS200
cg16091553	4	46126245	2	GABRG1	TSS200
cg02891314	5	179741120	2	GFPT2	Body
cg13944838	5	179740914	2	GFPT2	Body
cg00595030	19	10398582	2	ICAM4	3'UTR;Body;Body
cg19298774	19	10398798	2	ICAM4	3'UTR;3'UTR;Body
cg05323345	11	2163174	2	IGF2AS	Body;Body;5'UTR;TSS1500
cg21237591	11	2162510	2	IGF2AS	Body;Body;5'UTR;TSS200
cg18163342	14	105332627	2	KIAA0284	5'UTR;5'UTR
cg22454744	14	105332600	2	KIAA0284	5'UTR;5'UTR
cg03846641	2	109746751	2	LOC100287216	TSS200;Body
cg07548255	2	109746754	2	LOC100287216	TSS200;Body
cg12600858	11	92702530	2	MTNR1B	TSS1500
cg03970229	11	92702507	2	MTNR1B	TSS1500
cg04284140	7	44163975	2	POLD2	TSS1500;TSS1500
cg19084479	7	44163941	2	POLD2	TSS1500;TSS1500
cg03743584	10	135160908	2	PRAP1	1stExon;1stExon;5'UTR;5'UTR
cg15549126	10	135160910	2	PRAP1	1stExon;1stExon;5'UTR;5'UTR
cg05861697	17	1839990	2	RTN4RL1	Body
cg23289581	17	1840546	2	RTN4RL1	Body
cg12170787	19	1130965	2	SBNO2	Body;Body
cg18608055	19	1130866	2	SBNO2	Body;Body
cg08597832	8	144416327	2	TOP1MT	Body
cg23629150	8	144416404	2	TOP1MT	Body
cg23189410	3	147125712	2	ZIC4	TSS1500;TSS1500
cg06369327	3	147124523	2	ZIC4	TSS1500;TSS200
cg20119308	3	50376000	1	RASSF1	TSS1500;TSS1500;Body;Body

cg24893551	2	158453759	1	ACVR1C	Body;Body;5'UTR;Body
cg06010443	5	178594583	1	ADAMTS2	Body;Body
cg05160197	1	203135890	1	ADORA1	3'UTR;3'UTR
cg16417876	15	26109261	1	ATP10A	TSS1500
cg25446098	17	47209664	1	B4GALNT2	TSS1500;TSS200;TSS1500
cg18113332	11	368384	1	B4GALNT4	TSS1500
cg12873919	20	30309627	1	BCL2L1	Body;Body
cg12584394	19	2016427	1	BTBD2	TSS1500
cg07091481	10	82169149	1	C10orf58	5'UTR
cg05763097	14	103569340	1	C14orf73	Body
cg25929533	6	127840616	1	C6orf174	TSS200
cg20779964	20	54580070	1	CBLN4	TSS200
cg07583137	8	82644012	1	CHMP4C	TSS1500
cg13530039	11	62689557	1	CHRM1	TSS1500
cg03482866	13	111040579	1	COL4A2	Body
cg08572214	4	73936052	1	COX18	TSS1500
cg23495748	5	175223636	1	CPLX2	5'UTR;1stExon
cg04615859	17	7762123	1	CYB5D1	Body;TSS1500
cg05922210	4	155253864	1	DCHS2	Body;Body
cg00160914	1	112299263	1	DDX20	Body;TSS1500
cg20739526	7	154585989	1	DPP6	Body;Body;Body
cg18852618	3	113897736	1	DRD3	1stExon;5'UTR;1stExon;5'UTR
cg19341652	6	116600143	1	DSE	TSS1500;1stExon
cg19629120	22	43924705	1	EFCAB6	3'UTR;3'UTR
cg14276006	19	1289881	1	EFNA2	Body
cg15050051	7	1782000	1	ELFN1	5'UTR
cg18592365	1	79472536	1	ELTD1	TSS200
cg00926318	20	25177340	1	ENTPD6	5'UTR;Body
cg13389958	7	27280924	1	EVX1	TSS1500
cg06433816	9	140311437	1	EXD3	5'UTR
cg06290070	8	72274548	1	EYA1	TSS200
cg05385610	19	40421700	1	FCGBP	Body
cg07718903	20	6104261	1	FERMT1	TSS200
cg07565472	2	219848258	1	FEV	Body
cg06181697	3	50294603	1	GNAI2	Body;Body
cg14099468	1	235814814	1	GNG4	TSS1500;TSS1500
cg15133448	10	95327325	1	GPR120	Body
cg02011392	6	101847541	1	GRIK2	Body;Body;Body
cg03206445	9	140062197	1	GRIN1	3'UTR;3'UTR;Body
cg08616516	11	88796092	1	GRM5	5'UTR
cg10249705	11	106890150	1	GUCY1A2	TSS1500
cg10076968	14	74006090	1	HEATR4	5'UTR;Body
cg20022036	6	32549496	1	HLA-DRB1	Body
cg22370252	12	54446289	1	HOXC4	5'UTR;TSS1500
cg04771199	17	77311577	1	HRNBP3	5'UTR

cg23622369	17	40706682	1	HSD17B1	Body
cg04237608	1	18659049	1	IGSF21	Body
cg09932405	2	121107495	1	INHBB	3'UTR
cg17203063	12	75601711	1	KCNC2	Body;Body;Body
cg17412560	2	95963403	1	KCNIP3	Body
cg00012638	6	39280541	1	KCNK17	Body;Body
cg01385052	8	140643119	1	KCNK9	Body
cg25246084	19	4971487	1	KDM4B	5'UTR
cg21096966	14	45432458	1	KLHL28	TSS1500;1stExon
cg17633576	12	16726789	1	LMO3	Body;Body
cg14501219	17	62775713	1	LOC146880	Body;Body
cg11637682	6	147124984	1	LOC729176	TSS200;Body
cg15726169	6	53661064	1	LRRC1	Body
cg02646225	8	86020307	1	LRRCC1	Body;Body
cg06879998	8	20112334	1	LZTS1	Body
cg26112661	1	53704558	1	MAGOH	TSS1500
cg09696560	3	185178617	1	MAP3K13	Body
cg17796010	20	26188963	1	MIR663	TSS200
cg08739828	18	33768144	1	MOCOS	Body
cg16358155	3	195490169	1	MUC4	Body;Body;Body
cg00425764	16	15949616	1	MYH11	5'UTR;5'UTR;5'UTR;5'UTR
cg04100307	8	2034238	1	MYOM2	Body
cg07124642	17	39991813	1	NT5C3L	Body
cg25293325	17	873413	1	NXN	Body
cg07962315	9	77704473	1	OSTF1	Body;TSS1500;TSS1500
cg21501724	20	9818832	1	PAK7	5'UTR;5'UTR
cg25024993	5	140248610	1	PCDHA7	Body;Body;1stExon;5'UTR
cg03349953	5	140515207	1	PCDHB5	1stExon
cg18389639	10	97049610	1	PDLIM1	Body
cg16355231	1	2344979	1	PEX10	TSS1500;TSS1500
cg04153991	3	111577705	1	PHLDB2	TSS1500;Body;TSS1500
cg23548151	12	130823838	1	PIWIL1	5'UTR
cg13857646	9	140356757	1	PNPLA7	Body;Body
cg19526455	17	37787862	1	PPP1R1B	Body;Body
cg26529371	19	12911983	1	PRDX2	Body;Body
cg05696092	19	50084283	1	PRRG2	TSS1500;TSS1500
cg20073313	8	52322009	1	PXDNL	Body
cg04370983	8	117885438	1	RAD21	5'UTR
cg20920983	15	77224987	1	RCN2	Body
cg14486477	12	117256672	1	RNFT2	Body;Body
cg16767880	17	113262	1	RPH3AL	Body
cg07917609	5	33938225	1	RXFP3	1stExon
cg24602499	8	27765868	1	SCARA5	Body
cg09552652	16	23197569	1	SCNN1G	5'UTR
cg15829116	22	30901876	1	SEC14L4	TSS200;TSS200

cg17477442	17	27331600	1	SEZ6	Body;Body
cg01081083	22	25202766	1	SGSM1	Body;Body;Body;Body
cg06708634	6	100906895	1	SIM1	Body
cg02655630	20	44658941	1	SLC12A5	Body;Body
cg19421368	20	45280288	1	SLC13A3	TSS200;5'UTR
cg02707854	19	17600122	1	SLC27A1	Body
cg13716321	9	140130136	1	SLC34A3	Body
cg09204187	2	130940668	1	SMPD4	TSS1500;TSS1500;Body
cg22327175	11	121323934	1	SORL1	Body
cg02156870	17	36717733	1	SRCIN1	Body
cg20087519	10	135379242	1	SYCE1	TSS200;TSS200;5'UTR
cg15323840	6	158404061	1	SYNJ2	Body
cg13563634	6	32805684	1	TAP2	Body;Body
cg12928471	20	61492204	1	TCFL5	Body
cg07890553	3	119182858	1	TMEM39A	TSS1500
cg07645761	16	2892518	1	TMPRSS8	Body
cg14601038	2	1481097	1	TPO	Body;Body;Body;Body
cg20992663	1	115055124	1	TRIM33	TSS1500;TSS1500
cg23427912	4	189059510	1	TRIML1	TSS1500
cg22531668	10	118891601	1	VAX1	3'UTR
cg22698629	1	20670730	1	VWA5B1	Body
cg00374263	14	75229370	1	YLPM1	TSS1500
cg19913551	19	3806615	1	ZFR2	Body
cg07948034	13	100635905	1	ZIC2	Body
cg07778983	19	56905383	1	ZNF582	TSS1500
cg18693673	19	58629975	1	ZSCAN18	TSS200;TSS200
cg23591140	10	57389301			
cg25985355	7	65971099			
cg09208331	21	44864004			
cg16021678	13	55475951			
cg14462670	17	1363732			
cg13176198	15	30336915			
cg26613361	16	31830668			
cg24114014	6	28584167			
cg27026202	2	235200560			
cg05924485	19	22990378			
cg16291880	6	50674399			
cg03446244	16	86229872			
cg13998223	9	98389082			
cg10079374	7	158750417			
cg06635832	5	154654216			
cg02030219	16	1958654			
cg14283758	4	299370			
cg22797031	1	170630070			
cg23829948	4	3288458			

cg04253954	16	1148837			
cg19743545	2	237085808			
cg14582400	13	112726118			
cg26348995	17	80345367			
cg00983697	1	217314284			
cg13074055	14	106329206			
cg12823839	7	155175340			
cg05905179	15	73313741			
cg19498112	10	2951187			
cg26088680	14	95359766			
cg19460909	13	112515103			
cg16679630	8	70290423			
cg06197836	1	39283522			
cg15801340	19	38345819			
cg08727218	6	87832318			
cg17322044	12	125140105			
cg07694864	17	40700509			
cg18788932	12	115135700			
cg24729378	1	43609431			
cg09239150	12	132166381			
cg06224587	4	6540449			
cg13132370	17	46830260			
cg01389917	17	1956668			
cg24405999	3	133393119			
cg12764221	11	2883698			
cg16462006	14	95155784			
cg26279070	2	130971343			
cg01372551	7	98050564			
cg26098768	20	2187533			
cg10399099	16	51169072			
cg13692446	13	112759719			
cg03168896	3	44036098			
cg17842822	6	1310485			
cg26715559	6	30419491			
cg07856962	21	44864361			
cg03005851	1	11113287			
cg03296200	2	629022			
cg13420413	1	2347015			
cg04645150	6	28584172			
cg01624173	14	75981868			
cg20636352	2	200468832			
cg26405475	3	156324038			
cg26054764	1	214152805			
cg23875404	13	36728869			
cg02019125	3	156323952			

cg25288068	6	28793681			
cg22007638	1	3398886			
cg22481770	3	156324118			
cg03582285	17	40700314			
cg06246357	6	1524494			
cg02692390	5	172657465			
cg11556846	2	200468728			
cg09081997	13	51466941			
cg11115235	4	6247215			
cg03396347	1	1875803			
cg27083329	15	29210949			
cg23632393	1	2145116			
cg27049827	4	189376729			
cg14398295	15	30337018			
cg04117972	1	227635322			
cg19731055	19	5892313			
cg04651548	8	143085905			
cg04499083	1	151168668			
cg10779492	1	106623781			
cg22913127	19	34624975			
cg20893180	8	36957379			

Supplementary Table 7. Potential onco- and tumor suppressor genes differentially methylated in the CD34+ cells of *ASXL1* mutated patients.

Gene name	DNA methylation status in *ASXL1* mutated cases	Function
ARID5B	Hypomethylated in genebody	ARID5B is part of a H3K9me2 demethylase complex where it regulates transcription of target genes involved in cell growth and differentiation [2].
BCL6	Hypomethylated in the promoter region	BCL6 is a transcriptional repressor involved in several cellular processes including cell cycle progression, DNA damage response, and signal transduction [3, 4].
DIRC2	Hypomethylated in genebody	DIRC2 belongs to the major facilitator super family of transmembrane proteins responsible for transporting molecules across the cell membrane. Translocation of *DIRC2* is linked to renal carcinomas [5].
ICAM4	Hypomethylated in genebody	*ICAM4* encodes an adhesion receptor expressed by erythrocytes that can bind to several integrins found on endothelial cells [6], leukocytes [7, 8], and platelets [9].

miR-663	Hypermethylated in the promoter region	Mir-663 is among other biological processes involved in inflammatory response and carcinogenesis and shown to have a tumor suppressor function [10-12].
RABGAP1L	Hypermethylated in genebody	RABGAP1L is fused with MLL in leukemic cells [13] and involved in platelet dysfunction in a patient with *RUNX1* mutation [14].
RASSF1	Hypomethylated in the promoter region	RASSF1 plays a pivotal role in several pathways including cell cycle control, microtubule stabilization, cellular adhesion, cell motility, and apoptosis and has been suggested to have both a tumor suppressor- and oncogenic function [15, 16]
PRAP1	Hypomethylated in the promoter region	PRAP1 is linked to the survival of cancer cells in a p53 dependent manner as it promotes cell-cycle arrest after chemotherapeutic treatment [17]. PRAP1 has, in addition, recently been identified as a novel interacting partner of the mitotic arrest deficient 1 (MAD1) protein where *PRAP1* overexpression suppress mitotic checkpoint signaling in hepatocellular carcinoma [18].
TFAP2A	Hypermethylated in genebody	*TFAP2A* encodes a transcription factor that is involved in acute myeloid leukemia by inducing transcription of Hox genes and Meis1 [19].
TRPM2	Hypermethylated in genebody	*TRPM2* encodes a cation channel involved in apoptosis of hematopoietic cells [20] and is found to induce apoptosis in bladder cancer cells after histone deacetylase inhibition (HDACi) treatment [21].
TNXB	Hypomethylated in genebody	*TNXB* encodes an extracellular matrix glycoprotein with anti-adhesive function.

References

1. Gelsi-Boyer V, Trouplin V, Adelaide J, Bonansea J, Cervera N, Carbuccia N, Lagarde A, Prebet T, Nezri M, Sainty D *et al*: **Mutations of polycomb-associated gene ASXL1 in myelodysplastic syndromes and chronic myelomonocytic leukaemia**. *British journal of haematology* 2009, **145**(6):788-800.
2. Baba A, Ohtake F, Okuno Y, Yokota K, Okada M, Imai Y, Ni M, Meyer CA, Igarashi K, Kanno J *et al*: **PKA-dependent regulation of the histone lysine demethylase complex PHF2-ARID5B**. *Nature cell biology* 2011, **13**(6):668-675.
3. Shaffer AL, Yu X, He Y, Boldrick J, Chan EP, Staudt LM: **BCL-6 represses genes that function in lymphocyte differentiation, inflammation, and cell cycle control**. *Immunity* 2000, **13**(2):199-212.
4. Hatzi K, Jiang Y, Huang C, Garrett-Bakelman F, Gearhart MD, Giannopoulou EG, Zumbo P, Kirouac K, Bhaskara S, Polo JM *et al*: **A hybrid mechanism of action for BCL6 in B**

cells defined by formation of functionally distinct complexes at enhancers and promoters. *Cell reports* 2013, **4**(3):578-588.

5. Bodmer D, Eleveld M, Kater-Baats E, Janssen I, Janssen B, Weterman M, Schoenmakers E, Nickerson M, Linehan M, Zbar B *et al*: **Disruption of a novel MFS transporter gene, DIRC2, by a familial renal cell carcinoma-associated t(2;3)(q35;q21)**. *Human molecular genetics* 2002, **11**(6):641-649.
6. Zennadi R, Hines PC, De Castro LM, Cartron JP, Parise LV, Telen MJ: **Epinephrine acts through erythroid signaling pathways to activate sickle cell adhesion to endothelium via LW-alphavbeta3 interactions**. *Blood* 2004, **104**(12):3774-3781.
7. Bailly P, Tontti E, Hermand P, Cartron JP, Gahmberg CG: **The red cell LW blood group protein is an intercellular adhesion molecule which binds to CD11/CD18 leukocyte integrins**. *European journal of immunology* 1995, **25**(12):3316-3320.
8. Hermand P, Huet M, Callebaut I, Gane P, Ihanus E, Gahmberg CG, Cartron JP, Bailly P: **Binding sites of leukocyte beta 2 integrins (LFA-1, Mac-1) on the human ICAM-4/LW blood group protein**. *The Journal of biological chemistry* 2000, **275**(34):26002-26010.
9. Hermand P, Gane P, Huet M, Jallu V, Kaplan C, Sonneborn HH, Cartron JP, Bailly P: **Red cell ICAM-4 is a novel ligand for platelet-activated alpha IIbbeta 3 integrin**. *The Journal of biological chemistry* 2003, **278**(7):4892-4898.
10. Ni CW, Qiu H, Jo H: **MicroRNA-663 upregulated by oscillatory shear stress plays a role in inflammatory response of endothelial cells**. *American journal of physiology Heart and circulatory physiology* 2011, **300**(5):H1762-1769.
11. Zang W, Wang Y, Wang T, Du Y, Chen X, Li M, Zhao G: **miR-663 attenuates tumor growth and invasiveness by targeting eEF1A2 in pancreatic cancer**. *Molecular cancer* 2015, **14**:37.
12. Yan-Fang T, Jian N, Jun L, Na W, Pei-Fang X, Wen-Li Z, Dong W, Li P, Jian W, Xing F *et al*: **The promoter of miR-663 is hypermethylated in Chinese pediatric acute myeloid leukemia (AML)**. *BMC medical genetics* 2013, **14**:74.
13. Kowarz E, Burmeister T, Lo Nigro L, Jansen MW, Delabesse E, Klingebiel T, Dingermann T, Meyer C, Marschalek R: **Complex MLL rearrangements in t(4;11) leukemia patients with absent AF4.MLL fusion allele**. *Leukemia* 2007, **21**(6):1232-1238.
14. Sun L, Gorospe JR, Hoffman EP, Rao AK: **Decreased platelet expression of myosin regulatory light chain polypeptide (MYL9) and other genes with platelet dysfunction and CBFA2/RUNX1 mutation: insights from platelet expression profiling**. *Journal of thrombosis and haemostasis : JTH* 2007, **5**(1):146-154.
15. Agathanggelou A, Cooper WN, Latif F: **Role of the Ras-association domain family 1 tumor suppressor gene in human cancers**. *Cancer research* 2005, **65**(9):3497-3508.
16. Pelosi G, Fumagalli C, Trubia M, Sonzogni A, Rekhtman N, Maisonneuve P, Galetta D, Spaggiari L, Veronesi G, Scarpa A *et al*: **Dual role of RASSF1 as a tumor suppressor and an oncogene in neuroendocrine tumors of the lung**. *Anticancer research* 2010, **30**(10):4269-4281.
17. Huang BH, Zhuo JL, Leung CH, Lu GD, Liu JJ, Yap CT, Hooi SC: **PRAP1 is a novel executor of p53-dependent mechanisms in cell survival after DNA damage**. *Cell death & disease* 2012, **3**:e442.
18. Sze KM, Chu GK, Mak QH, Lee JM, Ng IO: **Proline-rich acidic protein 1 (PRAP1) is a novel interacting partner of MAD1 and has a suppressive role in mitotic checkpoint signalling in hepatocellular carcinoma**. *The Journal of pathology* 2014, **233**(1):51-60.
19. Ding X, Yang Z, Zhou F, Wang F, Li X, Chen C, Hu X, Xiang S, Zhang J: **Transcription factor AP-2alpha regulates acute myeloid leukemia cell proliferation by influencing**

Hoxa gene expression. *The international journal of biochemistry & cell biology* 2013, **45**(8):1647-1656.

20. Zhang W, Hirschler-Laszkiewicz I, Tong Q, Conrad K, Sun SC, Penn L, Barber DL, Stahl R, Carey DJ, Cheung JY *et al*: **TRPM2 is an ion channel that modulates hematopoietic cell death through activation of caspases and PARP cleavage**. *American journal of physiology Cell physiology* 2006, **290**(4):C1146-1159.
21. Cao QF, Qian SB, Wang N, Zhang L, Wang WM, Shen HB: **TRPM2 Mediates Histone Deacetylase Inhibition-Induced Apoptosis in Bladder Cancer Cells**. *Cancer biotherapy & radiopharmaceuticals* 2015, **30**(2):87-93.
